# Environmental and Clinical Strains of *Vibrio cholerae* Non-O1, Non-O139 From Germany Possess Similar Virulence Gene Profiles

**DOI:** 10.3389/fmicb.2019.00733

**Published:** 2019-04-12

**Authors:** Keike Schwartz, Jens Andre Hammerl, Cornelia Göllner, Eckhard Strauch

**Affiliations:** Department Biological Safety, German Federal Institute for Risk Assessment, Berlin, Germany

**Keywords:** *Vibrio cholerae*, North Sea, Baltic Sea, multilocus sequence typing, whole genome sequencing, virulence-associated factors, clinical isolates, Germany

## Abstract

*Vibrio cholerae* is a natural inhabitant of aquatic ecosystems globally. Strains of the serogroups O1 and O139 cause the epidemic diarrheal disease cholera. In Northern European waters, *V. cholerae* bacteria belonging to other serogroups (designated non-O1, non-O139) are present, of which some strains have been associated with gastrointestinal infections or extraintestinal infections, like wound infections or otitis. For this study, environmental strains from the German coastal waters of the North Sea and the Baltic Sea were selected (100 strains) and compared to clinical strains (10 isolates) that were from patients who contracted the infections in the same geographical region. The strains were characterized by MLST and examined by PCR for the presence of virulence genes encoding the cholera toxin, the toxin-coregulated pilus (TCP), and other virulence-associated accessory factors. The latter group comprised hemolysins, RTX toxins, cholix toxin, pandemic islands, and type III secretion system (TTSS). Phenotypic assays for hemolytic activity against human and sheep erythrocytes were also performed. The results of the MLST analysis revealed a considerable heterogeneity of sequence types (in total 74 STs). The presence of virulence genes was also variable and 30 profiles were obtained by PCR. One profile was found in 38 environmental strains and six clinical strains. Whole genome sequencing (WGS) was performed on 15 environmental and 7 clinical strains that were ST locus variants in one, two, or three alleles. Comparison of WGS results revealed that a set of virulence genes found in some clinical strains is also present in most environmental strains irrespective of the ST. In few strains, more virulence factors are acquired through horizontal gene transfer (i.e., TTSS, genomic islands). A distinction between clinical and environmental strains based on virulence gene profiles is not possible for our strains. Probably, many virulence traits of *V. cholerae* evolved in response to biotic and abiotic pressure and serve adaptation purposes in the natural aquatic environment, but provide a prerequisite for infection of susceptible human hosts. These findings indicate the need for surveillance of *Vibrio* spp. in Germany, as due to global warming abundance of *Vibrio* will rise and infections are predicted to increase.

## Introduction

The species *Vibrio cholerae* comprises Gram-negative bacteria which are distributed in aquatic ecosystems throughout the world. Strains of the serogroups O1 and O139 can cause the diarrheal disease cholera which affects millions of people in countries where the supply with clean drinking water is problematic. Poor sanitation as a result of damaged infrastructure occurring through natural catastrophes or human-caused disasters has led to cholera epidemics (Zuckerman et al., 2007). Major virulence factors of the toxigenic O1 and O139 strains are the cholera toxin (CTX) and the toxin-coregulated pilus (TCP), which are both encoded on mobile genetic elements integrated into the chromosome of the toxigenic strains (Harris et al., 2012). While the genes for CTX are part of the genome of the filamentous phage CTXΦ (McLeod et al., 2005; Bhattacharya et al., 2006), genes for the TCP biosynthesis are located within a pathogenicity island designated as *Vibrio* pathogenicity island VPI-1 (Karaolis et al., 1998; Murphy and Boyd, 2008).

However, most *V. cholerae* strains do not possess these two virulence factors and belong to other serogroups. On the basis of differences in the surface-expressed O antigen, more than 200 serogroups have been described and strains of these serogroups are commonly designated as *V. cholerae* non-O1, non-O139. A number of reports have been published revealing that some strains can cause intestinal infections and extraintestinal infections like wound and soft tissue infections, ear infections or bacteremia (Pang et al., 2007; Chatterjee et al., 2009; Octavia et al., 2013; Ceccarelli et al., 2015; Deshayes et al., 2015). There is no legal obligation to notify public health authorities of cases of non-O1, non-O139 *V. cholerae* infections in Germany so far, irrespective of the severity of the disease. In non-O1, non-O139 *V. cholerae* strains, a number of virulence factors can be present that are also found in the toxigenic strains and are known to contribute to the infection process in a synergistic way (Rivera et al., 2001; Singh et al., 2001). These accessory virulence factors include mannose-sensitive hemagglutinin pilus (MSHA), different hemolysins, repeats-in-toxin (RTX) toxin clusters, and outer membrane proteins (Schirmeister et al., 2014). However, the occurrence of these factors is diverse in the strains. More virulence factors are found only in some non-O1, non-O139 *V. cholerae* and contribute essentially to the pathogenicity of these strains. The type III secretion system (TTSS) was shown to be necessary for colonization and causing diarrheal disease in animal studies with pathogenic strains lacking the major virulence factors of the toxigenic strains (Dziejman et al., 2005; Shin et al., 2011).

Non-O1, non-O139 *V. cholerae* strains are present in marine environments of German coastal waters of the North Sea and Baltic Sea (Böer et al., 2013), whereas toxigenic *V. cholerae* strains do not occur in central Europe. Studies conducted in Austria revealed that Lake Neusiedl, a saline steppe lake, contained considerable numbers of non-O1, non-O139 *V. cholerae* bacteria. In other Austrian bathing waters, non-O1, non-O139 *V. cholerae* were also detected (Hirk et al., 2016). Phylogenetic analysis of the strains from Lake Neusiedl revealed a remarkable genomic diversity among these isolates and genetic similarities to strains from other European countries (Pretzer et al., 2017).

Climate change is known to have a great impact on *Vibrio* spp. occurrence in aquatic environments. Warming of water surface temperatures will lead to an increase of *Vibrio* spp. abundance and the incidence of human and animal infections caused by these bacteria is predicted to rise (Baker-Austin et al., 2012; Vezzulli et al., 2016). A number of infections and septicemia caused by non-O1, non-O139 *V. cholerae* and other *Vibrio* spp. have been reported by European countries of the Baltic Sea and North Sea region, although in Germany the number of infections is still quite low (Huehn et al., 2014). However, recent case reports of serious infections caused by non-O1, non-O139 *V. cholerae* from the Netherlands (Engel et al., 2016) and Austria (Hirk et al., 2016) emphasize the threat posed by these waterborne pathogens. Studies in Austria showed that temperature is the main predictor for *V. cholerae* abundance in lakes but the quantities of dissolved organic matter also influenced the *Vibrio* numbers (Bliem et al., 2018).

In Germany so far no studies in inland lakes were conducted. However, the occurrence of non-O1, non-O139 *V. cholerae* in marine and estuarine environments was already described several years ago (Bockemühl et al., 1986), although systematic studies were initiated only many years later in the framework of two research projects (Böer et al., 2013; Erler et al., 2015). These projects confirmed that such vibrios are indigenous to German coastal waters and their abundance underlies seasonal variations. A number of non-O1, non-O139 *V. cholerae* strains isolated in these projects was recently investigated for antimicrobial resistance (Bier et al., 2015). From this collection, we selected 50 strains from the North Sea region as well as 50 strains from the Baltic Sea for the current study in order to analyze the genetic relationships between the strains from the two seas. The Baltic Sea is an intracontinental ocean with low salinity, while the North Sea belonging to the Atlantic Ocean has reduced salinity near the estuarine regions. Therefore, *V. cholerae* strains of the North Sea were obtained only from recreational waters of the estuaries. In the current study, also clinical non-O1, non-O139 *V. cholerae* strains were included which came from patients who had contracted primarily extraintestinal infections from the coastal waters of Germany (Schirmeister et al., 2014).

By comparing MLST sequence types (STs) and virulence factor profiles, the aim of this study is to determine if environmental non-O1, non-O139 strains related to clinical strains are frequently present in the coastal waters of Germany. Rising water surface temperatures are expected to increase *Vibrio* abundance in future and data are needed for health authorities to carry out risk assessment for vibrios and to introduce measures to reduce the threat for the public.

## Materials and Methods

### Bacterial Strains

The non-O1, non-O139 *V. cholerae* strains used in this study (*n* = 110) are summarized in Table 1 and listed in detail in Supplementary Table S1. Environmental strains (*n* = 100) from the Baltic Sea (*n* = 50) and North Sea (*n* = 50) were isolated by health authorities and nationally recognized scientific institutions within the German research programs KLIWAS^1^ (*n* = 14) and VibrioNet^2^ (*n* = 86) between 2009 and 2014. Seawater and sediment samples were mostly collected at bathing sites along the Baltic Sea and North Sea coastline as well as within the estuaries of the rivers Ems and Weser (Böer et al., 2013). Bivalve mollusk samples came from coastal areas of the North Sea. Clinical strains (*n* = 10) were isolated from German patients with extraintestinal or intestinal infections between 1995 and 2017. Strains were obtained from the State Office for Health and Social Affairs (LAGuS), Rostock, and the Robert Koch Institute (RKI), Berlin, or from the strain collection of the German Federal Institute for Risk Assessment (BfR), Berlin. Most of the clinical isolates had been characterized by Schirmeister et al. (2014). All environmental and clinical isolates (except VN-00533 and VN-00534) had been tested for antimicrobial resistances in previous studies (Bier et al., 2015; Hammerl et al., 2017).

**Table 1:** Origin and source of *Vibrio cholerae* non-O1, non-O139 strains (*n* = 110) included in this study.

Origin	Geographical origin	Source	Source code
**Environmental** **(E)**	Baltic Sea (BS)	Seawater (sw)	E-BS -sw
(2009–2014; *n* = 100)	(*n* = 50)	(*n* = 37)	
		Sediment (sd)	E-BS -sd
		(*n* = 3)	
		Seawater/sediment (sw/sd)	E-BS -sw/sd
		(*n* = 10)	
	North Sea (NS)	Bivalve mollusks (bm)	E-NS -bm
	(*n* = 50)	(*n* = 26)	
		Seawater (sw)	E-NS -sw
		(*n* = 10)	
		Seawater/sediment (sw/sd)	E-NS -sw/sd
		(*n* = 14)	
**Clinical** **(C)**	Germany (G)	Extraintestinal (ext)	C-G -ext
(1995–2017; *n* = 10)	(*n* = 10)	(*n* = 8)	
		Intestinal (int)	C-G -int
		(*n* = 2)	

### Species Confirmation, Characterization, and Subtyping

Species confirmation of clinical and environmental *V. cholerae* strains was performed by whole-cell matrix-assisted laser-desorption/ionization time-of-flight mass spectrometry (WC-MALDI-TOF MS) analysis and PCR analysis as previously described (Schirmeister et al., 2014; Bier et al., 2015). For PCR analysis, genomic DNA was extracted from 1 ml of an overnight culture in lysogeny broth using the RTP Bacteria DNA Mini Kit according to the manufacturer’s protocol (Stratec Molecular GmbH, Berlin, Germany). Ten nanograms of genomic DNA served as template DNA. Primers, annealing temperature, and amplicon sizes of the *toxR*-*ctxA*-*rfb*O1-*rfb*O139 multiplex PCR assay are shown in Supplementary Table S2.

### Multilocus Sequence Typing (MLST)

Genetic relationships of the environmental isolates and clinical isolates (Supplementary Table S1) were characterized by *V. cholerae* specific MLST. Using the genomic DNA, MLST was performed targeting seven housekeeping genes (*adk*, *gyrB*, *mdh*, *metE*, *pntA*, *purM*, *pyrC*) (Octavia et al., 2013) and the ST was identified using the non-O1, non-O139 *V. cholerae* MLST scheme^3^. MLST primers, annealing temperatures, and amplicon sizes are shown in Supplementary Table S2. Sequences were assembled using the Lasergene software SeqMan Pro version 12.0 (DNASTAR Inc., Madison, WI, United States). The consensus sequences of each locus were aligned and trimmed to reference length in Accelrys Gene version 2.5 (Accelrys Inc., San Diego, CA, United States). New allele sequences and new allelic profiles were submitted to the PubMLST database for assignment of new allele numbers and ST numbers, respectively. To visualize genetic relationships between the isolates, allelic profiles were analyzed with the software PHYLOViZ version 1.1a using the goeBURST Full Minimum Spanning Tree (MST) algorithm (Francisco et al., 2012). A single-locus variant (SLV) comprised two STs differing at one locus only, while the other six loci were identical. A double-locus variant (DLV) included two STs differing at two out of the seven loci. A triple-locus variant (TLV) contained two STs differing at three housekeeping loci. Isolates showing 100% identity in at least six of the seven loci were assigned to a single clonal complex (CC), with at least two isolates forming the CC. To elucidate the genetic relationship between the *V. cholerae* isolates from German coastal waters and from German clinical samples, a full MST encompassing all identified STs was generated using PHYLOViZ software. Closer genetic relationships between the environmental and clinical isolates were visualized by the identification of CCs, SLVs, DLVs, and TLVs comprising STs of both origins. To describe the genetic diversity of the environmental strains, Simpson’s Index of Diversity *D* (Hunter and Gaston, 1988) was calculated for the strains from the Baltic Sea and North Sea using identified STs.

### Hemolytic Activity Tests

The hemolytic activity of the environmental isolates was determined based on the procedure given in Schwartz et al. (2017). Blood agar plates were prepared from Mueller-Hinton agar CM0337 (Oxoid GmbH, Wesel, Germany) supplemented with 4% human erythrocytes (German Red Cross, blood donation service, Berlin, Germany) or 4% sheep erythrocytes (BfR, Berlin, Germany). For semiquantitative investigation of the hemolytic activity of the strains, 10 μl of an overnight culture in Mueller-Hinton broth CM0405 (Oxoid) were spotted on a blood agar plate and incubated for 22–24 h at 37°C. The hemolytic activity was characterized based on the diameter of the hemolysis zone around the macrocolony (Supplementary Figure S1). Isolates were divided into four categories: non-hemolytic (-), weak hemolytic activity (+), intermediate hemolytic activity (++), and strong hemolytic activity (+++). Breakpoints for interpretation are given in Supplementary Table S3. The hemolytic activity tests were performed twice. Strains that gave equivocal results in two assays were retested in a third assay.

### PCR Typing of Virulence Genes

To recognize a possible pathogenic potential of the environmental isolates (Supplementary Table S1), the presence and absence of several virulence-associated genes and gene clusters was investigated according to Schirmeister et al. (2014). Prior to PCR typing of virulence-associated genes, bacterial strains were grown overnight and genomic DNA was extracted as described above. PCR amplification was performed in a total volume of 25 μl with 1 × PCR buffer (2 mM MgCl_2_), 0.2 mM of each dNTP, 1 μM of each primer, 2 U of DreamTaq DNA Polymerase (Thermo Fisher Scientific Biosciences GmbH, St. Leon-Rot, Germany), and 10 ng of template DNA. PCR reactions were performed using a Mastercycler ep gradient (Eppendorf AG, Hamburg, Germany). The PCR running conditions were as follows: an initial denaturation step at 94°C for 2 min (*hlyA*, *ompU*, *tcpA*), 4 min (*mshA*, *rstR*, *rtxC*, *chxA* (VC-Cholix-fo/-re), TTSS) or 5 min (*chxA* (VC-chxA-F/-R), *rtxA*, VSP-1, VSP-2), 30 cycles of denaturation at 94°C for 30 s (*hlyA* and *ompU* for 1 min, *tcpA* for 2 min), primer annealing for 30 s (*hlyA*, *ompU*, and *tcpA* for 1 min) and extension at 72°C for 1 min per kb, and a final extension step at 72°C for 10 min (*chxA* (VC-chxA-F/-R), *rtxA*, VSP-1, and VSP-2 for 7 min). PCR primers, annealing temperatures, and amplicon sizes are shown in Supplementary Table S2. To ensure the quality of the PCR system, negative (HPLC grade water) and external positive amplification controls (Supplementary Table S4) were used. Most PCR assays were performed in simplex formats. The presence of the genes *hlyA* (alleles Classical and El Tor) and *tcpA* (alleles Classical and El Tor) was examined by separate multiplex PCR assays. In the *hlyA* PCR, two universal *hlyA* primers (hlyA-489F, hlyA-1184R) and one El Tor *hlyA*-specific primer (hlyA-744F) were used. In the *tcpA* PCR, one universal *tcpA* forward primer (tcpA-F_Class-ET) and two allele specific *tcpA* reverse primers (tcpA-R_class, tcpA-R_ET) were utilized. Strains that gave negative results with *chxA* primers published by Awasthi et al. (2013) were retested with a newly designed primer pair (VC-Cholix-fo/-re). PCR products (1.5 μl each) were separated in GelRed stained agarose gels. Selected PCR products were purified and sequenced for confirmation.

### Whole Genome Sequence Determination and Bioinformatical Analysis

Whole genome sequence-based analyses were performed on a set of 22 non-O1, non-O139 *V. cholerae* strains. To gain deeper molecular insights into the genotypic traits of German environmental strains that are related to clinical strains in MLST STs, this included all SLVs, DLVs, and TLVs comprising both environmental and clinical STs (18 strains). In addition, four exemplary environmental strains showing an exceedingly low or high number of virulence-associated genes in PCR typing were selected for WGS-based analysis to confirm the PCR results and look for further virulence factors (Table 5). Preparation of genomic DNA and short-read whole genome sequencing (WGS; Illumina MiSeq, San Diego, CA, United States) was conducted as previously described (Schwartz et al., 2017). SPAdes *de novo* assemblies of raw reads were performed using the PATRIC database (release 3.5.21) (Wattam et al., 2017) following submission to the automated Prokaryotic Genome Annotation Pipeline of the NCBI website for genome annotation. Identification and assessment of putative prophage sequences was performed using the PHAge Search Tool (PHAST) according to the recommendations of the providers (Zhou et al., 2011). To detect specific genetic features within the genome sequences, different *in silico* analysis tools of the Center for Genomic Epidemiology (CGE), provided by the Danish Technical University, were used. Initial plasmid prediction was performed with the PlasmidFinder Web tool (release 2.0) (Carattoli et al., 2014). In addition, genomic contigs showing significantly higher sequence coverage levels than the rest of the contigs were screened for similarities to known plasmids using the BLASTN algorithm of the NCBI database^4^. For the initial detection of *Vibrio*-specific virulence determinants, the MyDbFinder Web tool (release 1.1) was used with a manually adapted database of the virulence factor database (VFDB^5^) (Chen et al., 2016). The database was derived from the VFDB DNA core dataset and only included *Vibrio* genes associated with experimentally verified virulence factors. CGE-based analyses were performed by using *de novo* assemblies of genomes. For *in silico* predictions, a minimum identity level of 50% (PlasmidFinder) and 30% (MyDbFinder), respectively, as well as a coverage level of at least 20% was used. To screen for *V. cholerae*-specific gene variants of the latter and of additional virulence determinants, the segmented genome fragments were applied to the BLASTN search of the NCBI database and compared to selected reference sequences using default settings. Accession numbers are given below. All isolates were analyzed for *V. cholerae*-specific sequences of *ompU*, *chxA*, *dth*, the NAG-ST gene (*stn*), *hapA*, *tlh*, *trh*, and *nanH*. *nanH*-positive isolates were further tested for the presence of genes of sialic acid metabolism enzymes as well as for the complete *Vibrio* pathogenicity island 2 (VPI-2) (Jermyn and Boyd, 2002). Isolates scored as potentially positive for sequences of the *Vibrio* seventh pandemic islands based on PCR results were screened for VSP-1 (Dziejman et al., 2002) and VSP-2 (O’Shea et al., 2004). All strains PCR-positive for the TTSS genes *vcsC2*, *vcsN2*, *vspD*, and *vcsV2* were further studied for the complete TTSS gene cluster (Dziejman et al., 2005; Chaand et al., 2015). In addition, *de novo* assemblies of the *mshA*-positive VN-00459 genome were analyzed for the presence of the MSHA gene cluster. To determine the phylogenetic relationship of the isolates, a CSI Phylogeny (version 1.4)-based SNP tree was prepared. The web-based tool was used under default settings and the exclusion of heterozygous SNPs. As reference genome, sequencing data of the clinical *V. cholerae* isolate VN-00533 (MWZM00000000) were used. Nucleotide variations were predicted according to the specifications provided by Kaas et al. (2014).

### Accession Numbers

Nucleotide sequences of new MLST alleles were deposited in *V. cholerae* PubMLST database sited at the University of Oxford under the numerical identifiers given in Supplementary Table S5. Genome sequences of *V. cholerae* isolates have been deposited in GenBank at the National Center for Biotechnology Information (NCBI) under the accession numbers given in Supplementary Table S9.

To screen the whole genome sequences for *V. cholerae*-specific variants of virulence determinants, reference sequences of virulence-associated genes and gene clusters were obtained from GenBank at NCBI. Accession numbers are CP000627.1 (*V. cholerae* O395; VC0395_A0162, *ompU*), NZ_GL989284.1 (*V. cholerae* BJG-01; VCBJG01_RS05475, *chxA*), NC_002506.1 (*V. cholerae* N16961; VCA1111, *dth*), M85198.1 (*V. cholerae* NRT-36; *stn*), CP028828.1 (*V. cholerae* N16961; N16961_VCA03457, *hapA*; N16961_VCA02936, *tlh*), NC_002505.1 (*V. cholerae* N16961; VC1784, *nanH*; VC1776-VC1779 and VC1781-VC1783, sialic acid metabolism homologs; VC1758-VC1809, VPI-2; VC0175-VC0185, VSP-1; VC0490-VC0516, VSP-2; VC0398-VC0411, MSHA cluster), AAKI03000011.1 (*V. cholerae* V51; VCV51_032643, *trh*), and DQ124262.1 as well as AATY02000000 (*V. cholerae* AM-19226; AATY02000003.1/AATY02000004.1, TTSS cluster and flanking regions).

## Results

### High Diversity of Strains Revealed by MLST

The data on allelic diversity and ST diversity are summarized in Supplementary Table S6 and listed in detail in Supplementary Table S5. Two *V. cholerae* strains from the Baltic Sea, VN-00455 and VN-00477, were excluded from data analyses due to failure of *pyrC* amplification.

In MLST analysis, a high number of new alleles and STs were found. The data revealed that all 108 strains (98 environmental strains and 10 clinical strains) possessed 74 different STs of which 71 were newly assigned STs based on the obtained sequencing data. The highest diversity of alleles was found in the *metE* and *pyrC* loci with 56 and 45 alleles, respectively. In the PubMLST database, these two loci show the highest genetic variability of the seven loci. The majority of STs (55) was present only once in each strain, 13 STs were found in two strains and only six STs in three or more strains. The genetic diversity is higher for strains of the Baltic Sea (*D* = 0.992; *N* = 48, *s* = 42) than for strains from the North Sea (*D* = 0.955; *N* = 50, *s* = 25). The STs of the 10 clinical strains included in this study also reveal a strong diversity between the strains (eight STs).

**Figure 1:**
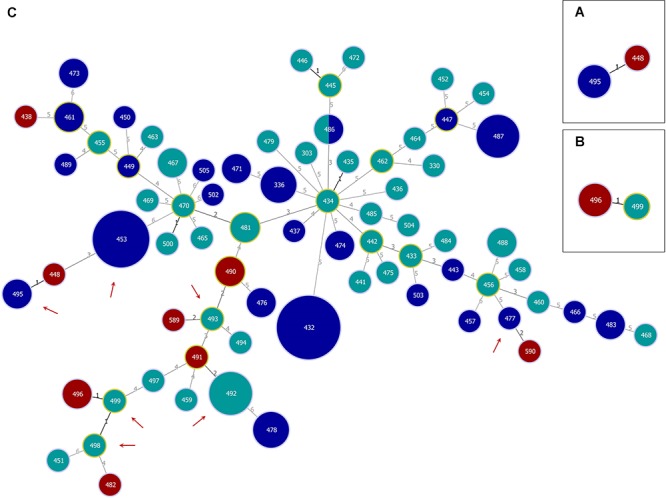
Genetic relationships of *Vibrio cholerae* non-O1, non-O139 isolates from German coastal waters and from German clinical samples obtained by goeBURST Full MST analysis based on MLST allelic profiles using PHYLOViZ 1.1a. Each sequence type (ST) is displayed as a circle with a size proportional to the number of isolates by which it is represented. The coloring indicates the origin: environmental/North Sea (dark blue), environmental/Baltic Sea (turquoise), and clinical/Germany (dark red). Circles surrounded by a green halo are (sub-) group founders. Single-locus variants (SLVs) are connected via black lines. Gray lines indicate connections with a higher level. The number of differing loci between two STs is shown next to the connection lines. Clonal complexes (CCs) formed at SLV level by North Sea and German clinical (CC1; **A**) or Baltic Sea and German clinical (CC2; **B**) isolates, respectively, as well as the full Minimum Spanning Tree (MST; **C**) are shown. In the full MST, environmental STs forming SLVs to TLVs (triple-locus variants) with clinical STs are marked with a dark red arrow.

The genetic relationships of the different subsets of strains were analyzed using the goeBURST Full MST algorithm (Figure 1). The goeBURST Full MST analysis visualized the clonal relations between all strains and revealed no clear separation between strains isolated from coastal waters of the North Sea and Baltic Sea regions. The analysis yielded two clonal complexes at SLV level comprising clinical and environmental strains (Figure 1A,B) and four clonal complexes consisting only of environmental strains (indicated by a solid black line, Figure 1C).

### Determination of Virulence Profiles by PCR

In a previous study (Schirmeister et al., 2014), we performed PCR genotyping to investigate the presence of virulence factors in clinical non-O1, non-O139 *V. cholerae* strains from German patients. The same set of primers was used to analyze the environmental non-O1, non-O139 strains with the aim to identify common virulence gene profiles between clinical and environmental strains.

Genes of the major virulence factors, the *ctxA* gene (CTX gene) and other elements of the CTX element (*zot*, *ace*, *rstR*) as well as the *tcpA* gene, were absent in both clinical and environmental *V. cholerae* non-O1, non-O139 strains (Supplementary Table S7). Besides the CTX element and the TcpA pilus, presence or absence of several factors was studied in the environmental strains. The genotyping of these virulence factors revealed a high diversity and a number of different virulence gene profiles were obtained.

In Table 2, the frequency of the presence of virulence factors is shown. The detailed results for every strain are shown in Supplementary Table S7. The *rtxC* gene and the *toxR* gene were the only genes detected in all strains. The second most abundant gene was the *hlyA* gene, as only in three strains of the same ST (ST336) no PCR products were visible. The products of the *hlyA* PCR displayed the El Tor variant of this gene (*hlyA*^ET^) (Rivera et al., 2001; Schirmeister et al., 2014). The PCR for the genomic islands VSP-1 and VSP-2 revealed the absence of the islands when a PCR product of the expected size is obtained. In approximately 80% of the strains, either VSP-1 or VSP-2 or both elements were missing. In approximately 75% of the strains, the *ompU* PCR was positive. The primers of the *rtxA* PCR were designed to detect the MARTX toxin of the O1 reference strain N16961 which was only found in 22% of all strains. Four genes of the TTSS were detected in only 7% of all strains, whereas the cholix toxin gene (*chxA*) was found in 22% of the strains. The comparison of the distribution of virulence factors between North Sea and Baltic Sea strains did not show noteworthy differences.

**Table 2:** Presence/absence of virulence-associated genes and gene clusters in *Vibrio cholerae* non-O1, non-O139 isolates from German coastal waters based on PCR data.^a^

Origin	Virulence-associated genes and gene clusters														
	*rfb* O1	*rfb* O139	*ctxA*	*toxR*	*tcpA*^b^	*rstR*^c^	VSP-1 absent^d^	VSP-2 absent^d^	*hlyA*^e^	*mshA*	*ompU*	*rtxA*^f^	*rtxC*	*chxA*	TTSS^g^
Environmental (*n* = 100)	0%	0%	0%	100%	0%	0%	79%	80%	97%	6%	75%	22%	100%	22%	7%
Baltic Sea (*n* = 50)	0%	0%	0%	100%	0%	0%	80%	86%	100%	6%	74%	28%	100%	14%	8%
North Sea (*n* = 50)	0%	0%	0%	100%	0%	0%	78%	74%	94%	6%	76%	16%	100%	30%	6%

^a^*The analysis also includes isolates with weakly positive PCR results. ^*b*^Alleles Classical and El Tor. ^*c*^Alleles Classical, El Tor, and Calcutta. ^*d*^The absence of VSP-1 and VSP-2 was demonstrated by the generation of 1.7 kbp and 800 bp PCR products, respectively, as the primers bind to flanking genomic sites of the pandemic islands. The generation of 3 kbp to 4 kbp PCR products indicated the presence of short VSP-1 fragments, while most of the pandemic island was absent. Strains showing none of the abovementioned PCR products were scored as potentially positive for VSP-1 and VSP-2. ^*e*^Specific for *V. cholerae* El Tor *hlyA*. All *V. cholerae* strains were negative for Classical *hlyA*. ^*f*^VC1451 of *Vibrio cholerae* O1 biovar El Tor str. N16961. ^*g*^The presence of TTSS was demonstrated by the detection of *vcsC2*, *vcsN2*, *vspD*, and vcsV2*.

A summary of all combinations of virulence factors obtained by PCR is shown in Table 3. We obtained a total of 30 profiles by PCR of which two profiles were also present in the German clinical strains. Strains were assigned to a different profile when no PCR products were obtained by using primers targeting the borders of VSP-1 and VSP-2 elements. One profile showed by far the highest frequency and was detected in 38 of the environmental strains (*toxR hlyA*^ET^ *rtxC ompU* positive, absence of VSP-1 and VSP-2) and in six clinical strains.

**Table 3:** Virulence gene profiles of *Vibrio cholerae* non-O1, non-O139 isolates from German coastal waters compared to virulence genotypes of clinical non-O1, non-O139 isolates from German patients based on PCR data and profiles further analyzed based on WGS data.

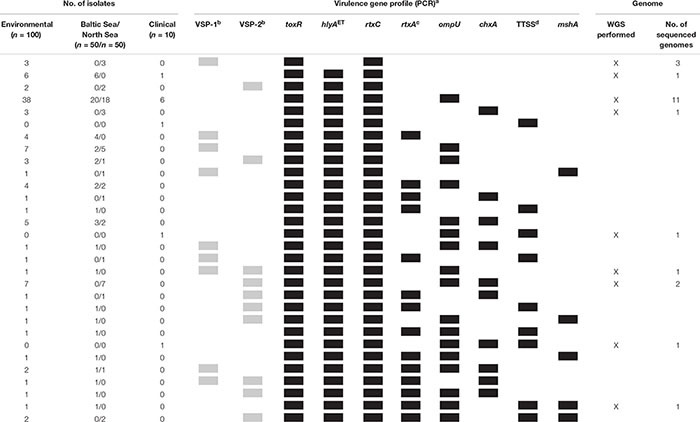

*ET, El Tor. ^*a*^All isolates were negative for *ctxA*, *tcpA* (Classical, El Tor), *rstR* (Classical, El Tor, Calcutta) and *hlyA* (Classical) PCR. A gray/black box indicates the potential presence/presence of the marker. ^*b*^The absence of VSP-1 and VSP-2 was demonstrated by the generation of 1.7 kbp and 800 bp PCR products, respectively, as the primers bind to flanking genomic sites of the pandemic islands. The generation of 3 kbp to 4 kbp PCR products indicated the presence of short VSP-1 fragments, while most of the pandemic island was absent. Strains showing none of the abovementioned PCR products were scored as potentially positive for VSP-1 and VSP-2. ^*c*^VC1451 of *Vibrio cholerae* O1 biovar El Tor str. N16961. ^*d*^The presence of TTSS was demonstrated by the detection of *vcsC2*, *vcsN2*, *vspD*, and *vcsV2*.*

### Hemolytic Activity Against Human and Sheep Erythrocytes

Hemolysis zones of strains were investigated on blood agar plates containing either sheep or human erythrocytes. The results of the hemolytic activity tests are summarized in Table 4 and listed in detail in Supplementary Table S8. The vast majority of the strains showed hemolytic activity against both types of erythrocytes. Human erythrocytes were more susceptible to some strains than sheep erythrocytes. One strain was not hemolytic against sheep erythrocytes but showed hemolysis on human blood cells. Only three strains showed no lytic activity for both types of blood cells. The three strains belonged to the same ST (ST336) and did not yield any *hlyA* PCR products.

**Table 4:** Hemolytic activity of *Vibrio cholerae* non-O1, non-O139 isolates from German coastal waters analyzed in this study.

Type of erythrocytes	Environmental				Baltic Sea				North Sea			
	(*n* = 100)				(*n* = 50)				(*n* = 50)			
	-	+	++	+++	-	+	++	+++	-	+	++	+++
Sheep	4%	26%	60%	10%	2%	26%	60%	12%	6%	26%	60%	8%
Human	3%	7%	71%	19%	0%	6%	78%	16%	6%	8%	64%	22%

*The percentage of strains showing no (-), weak (+), intermediate (++), or strong (+++) hemolysis against sheep erythrocytes and human erythrocytes, respectively, is given*.

### Whole Genome Sequencing (WGS)

The obtained genome sizes of the 22 sequenced strains vary between 3.9 and 4.2 Mbp and the average number of coding genes is approximately 3.74 × 10^3^. Detailed information about the genomes is given in Supplementary Table S9. The results of the bioinformatic analysis concerning the presence of virulence genes are shown in Table 5.

**Table 5:** Virulence gene profiles of *Vibrio cholerae* non-O1, non-O139 isolates from German coastal waters and from German clinical samples based on WGS data.

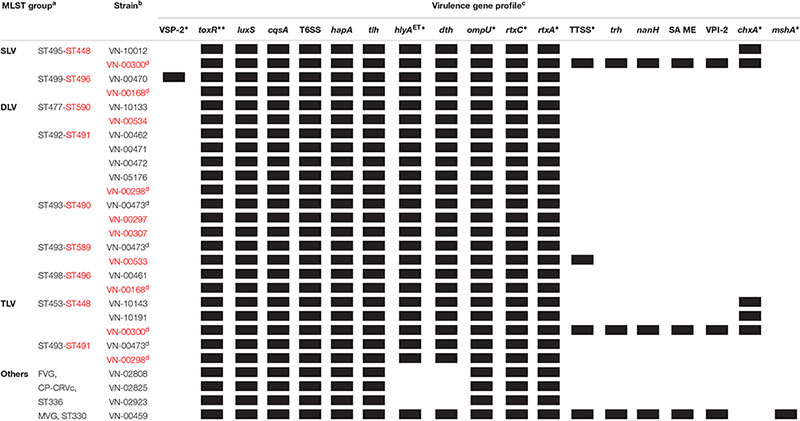

*CP-CRVc, carbapenemase producing carbapenem-resistant *Vibrio cholerae*; ET, El Tor; FVG, few virulence-associated genes/gene clusters (according to PCR data); MVG, many virulence-associated genes/gene clusters (according to PCR data); SA ME, sialic acid metabolism. ^*a*^The MLST groups are explained in section “Materials and Methods”. Clinical STs are marked in red. ^*b*^All strains of the MLST groups selected for whole genome sequencing were compared regarding sequence type, year of isolation, geographical origin, sample type, and PCR-based virulence gene profile. Presumably identical strains were summarized in subgroups. One strain from each subgroup was selected for whole genome sequencing. Clinical strains are marked in red. ^*c*^All strains were negative for VSP-1 and *stn*. Reference sequences for WGS analysis are given in section “Materials and Methods”. In general, strains showing >90% sequence similarity to a specific reference sequence were defined as positive for the respective virulence-associated trait. Further details are given in the “Results” section. ^∗^Virulence-associated gene/gene cluster initially investigated by PCR. ^∗∗^Virulence-associated gene only investigated by PCR. ^*d*^Strain belonging to more than one MLST group*.

The *hlyA*^ET^ gene is present in all strains with the exception of the three strains that were hemolysis negative, showing that the gene was absent in these strains. Another hemolysin gene (*dth*) encoding the delta thermostable hemolysin (Fallarino et al., 2002) is also absent in the three strains, but is found in the remaining strains.

The *rtxC* gene was detected in all strains confirming the PCR results. The PCR targeting the *rtxA* gene was positive only in one strain (VN-00459). The applied primers were designed to amplify an internal fragment containing the ACD region (actin cross-linking domain) of the *rtxA* gene encoding the multifunctional autoprocessing repeats-in-toxin toxin (MARTX) of the O1 reference strain N16961 (gene VC1451) (Sheahan et al., 2004). The bioinformatic analysis confirmed that this region of the *rtxA* gene was highly similar to the corresponding region of gene VC1451 only in strain VN-00459. All other sequenced strains also harbor *rtxA* genes encoding MARTX toxins that differ in effector domains encoded in the central part of the *rtxA* gene (Satchell, 2015).

We observed that all strains possess *ompU* genes. In five of the sequenced strains which were negative in the *ompU* PCR (Supplementary Table S7) the absence of a PCR product can be explained by sequence variations in the primer binding sites. The environmental strains VN-02808, VN-02825, and VN-02923 possess an *ompU* variant which is only 80% similar to the *ompU* gene of the *V. cholerae* O1 strain from which the primers were derived (accession NC_009457, Singh et al., 2001). The bioinformatic analysis also revealed that in four strains, VN-00300, VN-10012, and two closely related environmental strains (VN-10143, VN-10191), the cholix toxin encoding *chxA* gene is present confirming the PCR results.

In some strains, no PCR product was obtained when using primer targeting sequences to the left and right of the seventh pandemic islands VSP-1 and VSP-2. Therefore, it was checked if parts of the islands might be present in the genomes. Four strains (VN-00470, VN-02808, VN-02825, VN-02923) did not yield a PCR product of the expected size of 1.7 kbp if VSP-1 is missing (Rahman et al., 2008; Schirmeister et al., 2014). There were additional sequences enlarging the size of the fragment to 3.8 kbp in VN-00470, but no sequences of the VSP-1 island were detected in this strain nor in the other three strains.

**Figure 2:**
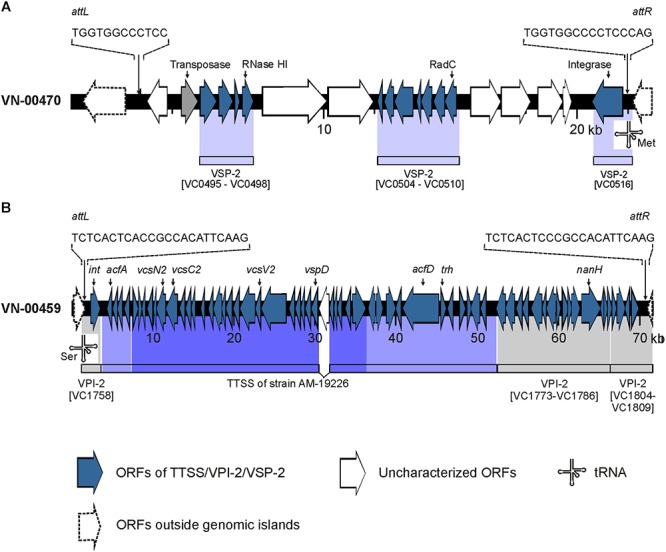
Schematics of genomic islands of the *Vibrio cholerae* non-O1, non-O139 strains VN-00470 and VN-00459 from the German Baltic Sea. **(A)** *Vibrio* seventh pandemic island 2 (VSP-2)-like element identified in strain VN-00470. Light blue areas below the VN-00470 sequence indicate similarity to genes of VSP-2 of *V. cholerae* O1 reference strain N16961 (gene numbering according to NC_002505.1). **(B)** Variant *Vibrio* pathogenicity island 2 (VPI-2) present in strain VN-00459. Gray areas below the VN-00459 sequence indicate similarity to genes of VPI-2 of *V. cholerae* O1 reference strain N16961 (gene numbering according to NC_002505.1). Blue colored areas below the VN-00459 sequence indicate similarity to the type three secretion system (TTSS) of strain AM-19226 (accession number AATY02000000). Dark blue regions represent the core region of the TTSS. Light blue regions represent the 5′- and 3′-flanking regions of the TTSS. *attL*, left attachment site; *attR*, right attachment site; ORF, open reading frame.

The BLASTN search for VSP-2 sequences gave a different result. In strain VN-00470, a VSP-2-like element (Haley et al., 2014) with a size of approximately 19.3 kbp was detected that harbored homologs (similarity > 92%) of the genes VC0495–VC0498 and VC0504–VC0510 of the VSP-2 of strain N16961. Between these regions, a 4.3 kbp fragment is present that carries two coding sequences of proteins with uncharacterized functions (Figure 2A). The integration of the element is in the same position of the chromosome as described for VSP-2 of strain N16961. A homolog of the phage integrase gene of VSP-2 (VC0516) is located at the right border of the VSP-2-like element and *attL* and *attR* sequences highly similar to the corresponding sites of VSP-2 of N16961 were identified (Murphy and Boyd, 2008). Investigation of the sequence data of the strains VN-10143 and VN-10191 revealed that only a short sequence (ca. 900 bp) is similar to a pseudogene region of VSP-2 (VC0501) which could encode a functional transposase.

In strain VN-00459, the PCR for the *mshA* gene yielded a product of the expected size. An alignment of the gene cluster encoding the MSHA pilus of strain N16961 to the genomic sequences revealed that the cluster is present in this strain (similarity > 99%).

Four genes of the TTSS of non-O1, non-O139 strains (Dziejman et al., 2005; Chaand et al., 2015) were detected by PCR in three strains used for WGS (VN-00300, VN-00533, VN-00459) (Supplementary Table S7). The bioinformatic analysis makes it likely that the complete TTSS cluster consisting of core region and 5′- and 3′-flanking regions of reference strain AM-19226 is present in strains VN-00300 and VN-00459 (Chaand et al., 2015). The cluster sequences possess a similarity of >95% to the TTSS of the reference and a *trh* gene encoding a thermostable direct hemolysin is also found in the 3′-flanking region (Figure 2B). The TTSS of strain VN-00533 comprises only the core region and few genes of the 3′-flanking region excluding the *trh* gene and another putative virulence gene (*acfD*) (Chaand et al., 2015).

The *nanH* gene which encodes a neuraminidase cleaving sialic acid residues from host gangliosides (Almagro-Moreno and Boyd, 2009) were also found in the genomes of two TTSS harboring strains (VN-00300, VN-00459). As the *nanH* gene is part of the *Vibrio* pathogenicity island VPI-2 (Murphy and Boyd, 2008), an alignment of VPI-2 of strain N16961 was performed to the two genomes. In both strains, the genes of the sialic acid metabolism are present (VC1773–VC1786). In strain VN-00459, the TTSS and the sialic acid metabolism genes lie on the same contig and form a variant VPI-2 island with a size of 68.9 kbp (Figure 2B) (Murphy and Boyd, 2008). The element is integrated into the same chromosomal position as VPI-2 in the reference strain N16961 and possesses identical *attL* and *attR* sequences to VPI-2. In strain VN-00300, the variant VPI-2 sequences are distributed on several different contigs. However, the combined sizes of sequences and their high similarity to VN-00459 suggest that both strains harbor the same genomic island. In the third TTSS harboring strain, VN-00533, the TTSS sequences are adjacent to a truncated coding sequence (approximately 53%) of the *nanH* gene. This pseudogene is flanked by a 2.7 kbp sequence with high similarity to the right border of a VPI-2 element. However, no more sequences of a variant VPI-2 element are found.

The analysis also revealed that environmental and clinical strains are probably equipped with a functional type six secretion system (T6SS) as most of the genes of the core region and some effector proteins (genes *vipAB*, *vasA-vasK*, *vgrG-3*, *vgrG-2*) are present in all genomes (similarity > 95%) (Zheng et al., 2011; Unterweger et al., 2014).

The bioinformatic analysis was extended to include some virulence genes whose presence/absence has been studied in non-O1, non-O139 strains. The prevalence of the *stn* gene encoding the heat-stable enterotoxin (Ogawa et al., 1990) and the *tlh* gene encoding a heat-labile hemolysin (Syed et al., 2009), were analyzed. While the former gene was absent, the latter was found in all sequenced strains. In a process called quorum sensing, the expression of virulence genes of toxigenic *V. cholerae* strains is influenced by two major autoinducers CAI-1 and AI-2 (Higgins et al., 2007). The genes *cqsA* and *luxS* encoding synthases of the two autoinducers were found in all sequenced genomes. Another virulence gene, *hapA*, codes for the *V. cholerae* hemagglutinin/protease that degrades host proteins of intercellular tight junctions (Wu et al., 2000). The gene was also detected in all strains.

PCR typing of virulence genes and whole genome sequence-based analyses revealed an overall matching degree of 84%. In 15% of cases, the virulence factor in question was only detected by WGS-based bioinformatical analyses. In 1% of cases, “positive” PCR results were not confirmed by WGS. This only concerned VSP-2 PCR assay, which involves primers binding to flanking regions of the genomic island and where strains are to be assessed as potentially VSP-2 positive when a PCR product of the expected size is absent.

### SNP Analysis of Sequenced Genomes

**Figure 3:**
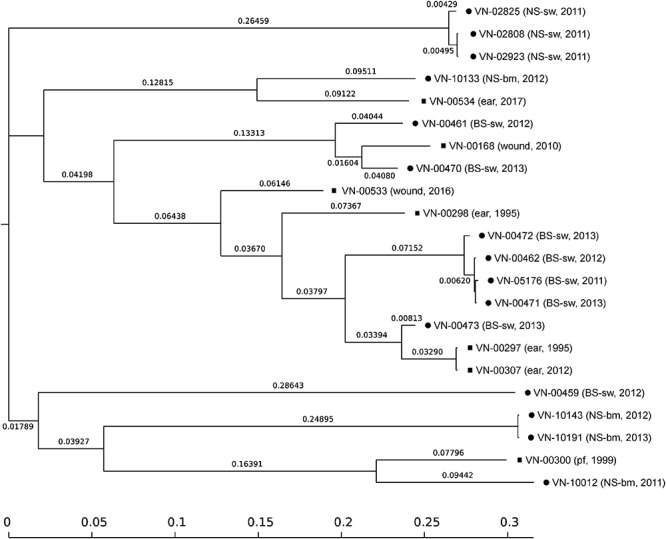
SNP-based phylogeny tree of *Vibrio cholerae* non-O1, non-O139 isolates from German coastal waters (•) and from German clinical samples (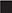). SNP tree was conducted using CSI Phylogeny 1.4 under default settings and the exclusion of heterozygous SNPs. Single nucleotide polymorphisms (SNPs) were called by mapping to the *V. cholerae* VN-00533 genome as reference (MWZM00000000). Scale bar represents the number of nucleotide substitutions per site and numbers indicate branch length. Basic information about the sample type and the year of isolation are given after the strain identifier: North Sea/seawater (NS-sw), North Sea/bivalve mollusk (NS-bm), Baltic Sea/seawater (BS-sw), and peritoneal fluid (pf).

All sequenced genomes were analyzed in an SNP analysis with the genome of strain VN-00533 as a reference. In total, 3,237,190 positions (corresponding to approximately 78.7% of the reference genome) were used in the analysis and the number of SNPs between the strains varied between 1 and 30,647 (Supplementary Table S10). The SNP tree is shown in Figure 3. Genomes of strains belonging to the same ST form clusters (SNP difference 1 to 847). Three clusters comprise only environmental strains (ST336 with strains VN-02808, VN-02825, VN-02923; ST492 with strains VN-00462, VN-00471, VN-00472, VN-05176; ST453 with the strains VN-10143 and VN-10191) and one cluster consisted of two clinical strains (ST490 with strains VN-00297 and VN-00307). The SNP tree shows that strains with single-locus variations in the MLST genes also group together, however, the SNP differences are distinctly higher. There are 11,892 SNPs between strains VN-10012 and VN-00300 and 4,657 between strains VN-00470 and VN-00168.

## Discussion

### MLST and Virulence Gene Profiles Obtained by PCR

As a basis for comparison, an MLST analysis was performed for all strains. MLST defines strains from the sequences at housekeeping loci and has become the method of choice for molecular typing of many bacterial species. Sequence data are ideal for strain characterization as they are unambiguous and strains can readily be compared between laboratories via the internet (Aanensen and Spratt, 2005).

Our study revealed a significant diversity of *V. cholerae* non-O1, non-O139 strains from the German coastal waters. High genetic diversity of *V. cholerae* non-O1, non-O139 strains has been also observed in other studies in which environmental strains were analyzed by MLST (Octavia et al., 2013; Pretzer et al., 2017; Siriphap et al., 2017). The STs of five German *V. cholerae* non-O1, non-O139 strains which were already deposited in the PubMLST database were distinctly different. All deposited STs had at most one common locus with the STs of the strains analyzed by Octavia et al. (2013). Interestingly, Chinese serogroup O1 strains which were lacking the *ctxAB* genes displayed a high MLST diversity in contrast to toxigenic O1 strains (Zhou et al., 2014). Though the applied MLST scheme only partially overlapped with the standardized PubMLST scheme, this indicates that high genetic diversity can also be found within a serogroup (Zhou et al., 2014).

As a next step, the presence or absence of virulence factors in the environmental strains was studied by PCR. While genes of the CTX element and the TcpA pilus were not detected in any of the strains, some accessory virulence genes of toxigenic strains which play a synergistic role in the infection process were found. The investigated factors were the MSHA (Rahman et al., 2008), the *rtxA* and *rtxC* genes of the RTX toxin cluster (Chow et al., 2001; Satchell, 2007), an outer membrane protein (OmpU) (Mathur et al., 2007), the virulence regulator gene *toxR* and the hemolysin gene *hlyA* (Rivera et al., 2001; Rahman et al., 2008; Chatterjee et al., 2009). Additionally, the occurrence of two genomic islands of toxigenic *V. cholerae* strains of the seventh pandemic (VSP-1, VSP-2) was examined (Murphy and Boyd, 2008). Some virulence factors like the TTSS and the cholix toxin (*chxA*) that are not associated with toxigenic strains were also investigated.

In a study of non-toxigenic *V. cholerae* serogroup O1 strains (Zhou et al., 2014), the virulence profiles differed among each other. A number of strains harbored the *Vibrio* pathogenicity island-1 (VPI-1) that encodes the TCP (Murphy and Boyd, 2008) and all strains possessed a MSHA pilus. We did not find VPI-1 genes in the non-O1, non-O139 strains and detected the *mshA* gene encoding the major pilin subunit (Marsh and Taylor, 1999) only in 6% of the strains.

In total, we obtained 30 profiles by PCR (Table 3) of which two profiles were also present in the German clinical strains (Schirmeister et al., 2014). The most frequent virulence gene profile comprised the genes *toxR*, *hlyA*^ET^, *ompU*, and *rtxC* and was found in 38 environmental and in six clinical strains.

It is remarkable that so many clinical strains belong to the group containing relatively few virulence genes. This could mean that many environmental strains with this profile could be potential human pathogens. While this conclusion seems plausible it also indicates the need for a deeper molecular analysis of the genetic makeup of the strains. For this reason, a number of strains with this profile were selected for WGS (see section “WGS Analysis of Virulence Factors and SNP Analysis”).

### Hemolytic Activity and Hemolysin Genes

Hemolysis of erythrocytes is a virulence trait widely distributed among pathogenic *Vibrio* species (Zhang and Austin, 2005) and is a phenotype that is routinely investigated in laboratories. We studied hemolytic activity of the strains against human and sheep erythrocytes to find out if the presence of the El Tor hemolysin gene is correlated to the lytic activity. Strains of the classical biotype carry a truncated hemolysin gene and do not lyze sheep erythrocytes (Alm et al., 1988). In a previous study (Rivera et al., 2001) it was observed that most non-O1, non-O139 strains possessed the El Tor *hlyA* gene, while few strains contained the classical *hlyA* gene with a premature stop codon and few strains lacked the gene.

The investigated environmental strains of our study possessed the *hlyA*^ET^ gene and were hemolytic on both types of blood cells. One strain showed hemolysis only on human cells but was not further studied. Three strains which had been negative in the *hlyA* PCR did not show hemolytic activity against both types of blood cells. This result could also be explained either by absence of the gene in the three strains or by PCR failure due to sequence variations to the applied primers. As *hlyA* has been used as a species marker for *V. cholerae* (Fykse et al., 2007; Jones et al., 2014) the three strains were included in the whole genome analysis and the absence of the gene was confirmed.

Remarkably, the *dth* gene related to the *δ-vph* gene of *V. parahaemolyticus* (Fallarino et al., 2002) was another hemolysin gene missing in the three strains, while detected in all remaining strains. However, it is not clear if under the tested conditions this gene contributes to the hemolytic activity at all. The *tlh* gene encodes a thermolabile hemolysin whose role in infection process is unclear though it was found to be upregulated in flagellar regulatory mutants in the same way as other virulence genes (Syed et al., 2009). The bioinformatic analysis showed that all strains possess this gene.

### WGS Analysis of Virulence Factors and SNP Analysis

The WGS data were used to confirm the PCR results and to identify more virulence genes in the genomes of the strains. In the context of the bioinformatical analysis, the majority of PCR results (84%) were confirmed. In some strains that had shown negative results in the *ompU* PCR (5 strains) or the *rtxA* PCR (21 strains) in consequence of primer mismatches, variants of the virulence genes were found using WGS data. This underlines the higher resolving power of WGS-based analytical methods and emphasizes the need for completion of PCR-based virulence typing schemes by WGS-based analyses for a risk assessment of bacterial isolates.

To determine the genetic relationship of the sequenced strains in more depth, an SNP analysis was performed. The SNP tree (Figure 3) shows that strains of the same STs are closely related, whereas a clear separation in respect of SNP numbers is found between strains which differ only in one of the seven housekeeping loci used for MLST. The SNP tree also clearly reveals that most strains are not closely related with the exceptions of strains of the same ST. Mostly, more than 20,000 SNPs are calculated between the strains.

The bioinformatic analysis confirmed that in the German strains no sequences of the CTX element and the TCP genes are present. In other studies, few non-O1, non-O139 strains were described that harbored *ctx*, *ace*, *zot*, or *tcpA* genes (Chatterjee et al., 2009; Ceccarelli et al., 2015).

A number of virulence genes present in all strains encode proteins that are probably primarily important for survival and niche adaptation in the natural aquatic environment (Sakib et al., 2018). An example is the *hapA* gene encoding a hemagglutinin/protease that affects epithelial tight junctions and contributes to diarrheal disease (Wu et al., 2000). In the natural environment, this protein is controlled by quorum sensing and degrades egg masses of insects (chironomids) which are a natural reservoir for *V. cholerae* (Halpern, 2010). Therefore, genes like *luxS* or *cqsA* encoding proteins involved in quorum sensing (Higgins et al., 2007) are also found in all genomes. The list of genes which are probably more important for survival in the natural aquatic environment rather than in a human host could be continued and explains why a number of virulence genes investigated in this study are present in all environmental strains (Sakib et al., 2018).

Another example is probably the *rtx* gene cluster. All strains harbor the *rtxC* gene encoding an acyltransferase which has been described as an activator of MARTX toxin. The role of the acyltransferase in infection is unclear, as the *rtxC* gene product is not necessary for MARTX function in an animal model (Cheong et al., 2010). While the *rtxC* gene is highly similar (identity > 91%) in all strains, the *rtxA* genes encoding MARTX vary considerably. MARTX proteins of *V. cholerae* strains are very large multifunctional proteins (4,565 amino acids in the toxigenic O1 strain N16961) with conserved N-terminal and C-terminal regions, whereas the central part of the toxin carries different effector domains (Satchell, 2015). The MARTX toxin of toxigenic El Tor strains possess three conserved internal domains and is probably involved in evasion of the host immune defense rather than in contributing to diarrheal disease.

In one sequenced strain (VN-00459) that was positive in the *rtxA* PCR a toxin gene highly similar to MARTX of the toxigenic O1 strain N16961 is present (identity of amino acid >98%). This MARTX toxin could be present in 21 more environmental strains that were positive in the *rtxA* PCR (Supplementary Table S7). With the exception of VN-00459, the remaining sequenced strains (clinical and environmental strains) harbored *rtxA* genes encoding MARTX variants differing in the central part of the coding region. It is known that environmental non-O1, non-O139 *V. cholerae* strains carry MARTX variants with different effector domains. These toxins can be active against eukaryotic cells from different organisms (mammals or fish or eels) and could play a role in adaptation to specific niches in the natural ecosystem (Satchell, 2015; Sakib et al., 2018).

The bioinformatic analysis of the genomes revealed that all strains probably harbor a functional T6SS. T6SS is a contact-dependent contractile apparatus resembling the spiked tail and tube of bacteriophages and is used for translocation of effector proteins mediating antagonistic interactions against many prokaryotic and eukaryotic organisms (Joshi et al., 2017; Sakib et al., 2018). In the environment, it protects against predators and helps in competition against antagonistic microorganisms and provides growth advantages. In diarrheal disease, it contributes to intestinal colonization against the gut microbiota and delivers effectors that are associated with intestinal inflammation and diarrheal symptoms in animal models. The role of T6SS in the environment and in infections explains why the secretion system is found in all clinical and environmental strains.

With the WGS sequence data, the genetic structure of islands and clusters detected by PCR in only few strains could be analyzed. The four TTSS genes as well as the *mshA* gene were identified as members of complete gene clusters encoding probably functional virulence factors. The MSHA pilus contributes to adhesion to cell surfaces and biofilm formation in the natural aquatic ecosystem (Chiavelli et al., 2001; List et al., 2018) while its role in human disease is not clear. The major pilin subunit gene *mshA* was present only in one of the sequenced strains. The TTSS is located within a variant VPI-2 genomic island together with more virulence factors (neuraminidase and enzymes of the SA metabolism). VPI-2 was shown to be a mobile genetic element (Murphy and Boyd, 2008).

Genes of the VSP-1 island are absent in all strains. However, in case of VSP-2, in one strain (VN-00470) a VSP-2-like element was identified (Haley et al., 2014). It is not clear if this element can contribute to virulence in humans. Similar elements are present in non-pathogenic *Vibrio* species indicating a role in the natural environment (Haley et al., 2014).

It is also possible that variations in promoter regions influence the expression of virulence genes. This could result in different manifestations of non-O1, non-O139 *V. cholerae* infections. In some *V. parahaemolyticus* strains harboring two hemolysin genes (*tdh*1 and *tdh*2), one gene product (TDH2) is found predominantly in culture supernatants. Analyses of the promoter sequences revealed differences in the -35 and -10 promoter sequences (Nishibuchi and Kaper, 1990; Nishibuchi et al., 1991). The question if variations in promoter sequences contribute to gene expression and hence influence the virulence of a strain can only be addressed in experimental studies.

## Conclusion

The aim of our study was to determine if STs and virulence gene profiles of clinical and environmental *V. cholerae* non-O1, non-O139 strains originating from German coastal waters may be correlated and could be usable for a risk assessment of individual strains. The result of this study reveals that – given on the current knowledge about the potential pathogenicity of these strains – no such correlation is found. The MLST shows a high diversity of the bacteria, whereas a basic equipment with virulence genes is very similar between all strains. Due to horizontal gene transfer, in some strains the number of virulence genes is increased compared to other strains. TTSS harboring *V. cholerae* non-O1, non-O139 strains were discovered in diarrhea causing strains (Dziejman et al., 2005) and recent studies have confirmed the importance of these systems for gastrointestinal infections (Chaand et al., 2015). The clinical strains of our study were mostly isolated from extraintestinal infections (wound infection and otitis) and lacked the genes of the TTSS system with one exception (strain VN-00533). This suggests that other virulence factors play a role in this type of infections.

*Vibrio cholerae* is a species that is a natural inhabitant of aquatic ecosystems. It is likely that most of the factors that contribute to virulence in a human host evolved as a response of these bacteria to challenges in their natural environment (Sakib et al., 2018). Thus, the genetic makeup of these bacteria is a result of biotic and abiotic stress in aquatic environments and adaptation to specific niches. This means that in the group of non-O1, non-O139 strains many of the so far recognized virulence genes are found in most isolates. It also means that a clear distinction between clinical and non-pathogenic environmental strains is probably not possible. The expected rise of *Vibrio* abundance in German coastal waters and the predicted increase of infections with these bacteria due to climate change demand measures and actions of the health authorities to reduce the risk for the public. For the time being, this could be the build-up of monitoring systems for vibrios at popular resort beaches and the establishment of surveillance systems for *Vibrio* infections by introducing a compulsory notification for diseases caused by these bacteria.

## Author Contributions

KS and ES designed the study. KS and CG performed the experiments. KS, JH, and ES analyzed the data, prepared the tables and figures, and wrote the manuscript. All authors edited the manuscript.

## Conflict of Interest Statement

The authors declare that the research was conducted in the absence of any commercial or financial relationships that could be construed as a potential conflict of interest.

## Footnotes

1 https://www.kliwas.de/

2 http://www.vibrionet.de/

3 https://pubmlst.org/vcholerae/

4 https://www.ncbi.nlm.nih.gov

5 http://www.mgc.ac.cn/VFs/

## Supplementary Material

The datasets generated for this study can be found in PubMLST (Supplementary Table S5) and GenBank (NCBI; Supplementary Table S9).
