# Supplementary material for: Environmental and Clinical Strains of Vibrio cholerae Non-O1, Non-O139 From Germany Possess Similar Virulence Gene Profiles
Source: Front Microbiol. 2019 Apr 12;10:733. doi: 10.3389/fmicb.2019.00733 (PMC6474259; doi:10.3389/fmicb.2019.00733)
Supplement: Supplementary file 1 [file Table_1.pdf]

Table S1. ***Vibrio cholerae*** **non-O1, non-O139 strains from German coastal waters and from German clinical samples used in this study.**

Strain	Year of isolation	Geographical origin	Source	Source code[2]	Institutional origin[4]
**Environmental - Baltic Sea**					
VN-00278	2011	Lubmin	Seawater	E-BS-sw	LAGuS
VN-00455	2011	Greifswald Bodden	Seawater	E-BS-sw	LAGuS
VN-00456	2012	Stresow	Seawater	E-BS-sw	LAGuS
VN-00457	2012	Wohlenberg Wiek/Niendorf	Seawater	E-BS-sw	LAGuS
VN-00458	2012	Karlshagen	Seawater	E-BS-sw	LAGuS
VN-00459	2012	Lubmin	Seawater	E-BS-sw	LAGuS
VN-00460	2012	Lubmin	Seawater	E-BS-sw	LAGuS
VN-00461	2012	Karlshagen	Seawater	E-BS-sw	LAGuS
VN-00462	2012	Lubmin	Seawater	E-BS-sw	LAGuS
VN-00463	2013	Lubmin	Seawater	E-BS-sw	LAGuS
VN-00464	2013	Warnemünde	Seawater	E-BS-sw	LAGuS
VN-00465	2013	Dranske/Libben	Seawater	E-BS-sw	LAGuS
VN-00466	2013	Binz/Prora	Seawater	E-BS-sw	LAGuS
VN-00468	2013	Spandowerhagen Wiek/Freest	Seawater	E-BS-sw	LAGuS
VN-00469	2013	Karlshagen	Seawater	E-BS-sw	LAGuS
VN-00470	2013	Lubmin	Seawater	E-BS-sw	LAGuS
VN-00471	2013	Lubmin	Seawater	E-BS-sw	LAGuS
VN-00472	2013	Stresow	Seawater	E-BS-sw	LAGuS
VN-00473	2013	Karlshagen	Seawater	E-BS-sw	LAGuS
VN-00474	2013	Stresow	Seawater	E-BS-sw	LAGuS
VN-00475	2014	Wohlenberg Wiek/Niendorf	Seawater	E-BS-sw	LAGuS
VN-00476	2014	Karlshagen	Seawater	E-BS-sw	LAGuS
VN-00477	2014	Lubmin	Seawater	E-BS-sw	LAGuS
VN-02995	2011	Schönhagen	Seawater	E-BS-sw	AWI
VN-03901	2011	Greifswald Bodden	Seawater/sediment	E-BS-sw/sd	LAGuS
VN-03902	2011	Darss-Zingst	Seawater/sediment	E-BS-sw/sd	LAGuS
VN-03903	2011	Wohlenberg Wiek/Niendorf	Seawater	E-BS-sw	LAGuS
VN-03907	2011	Darss-Zingst	Seawater/sediment	E-BS-sw/sd	LAGuS
VN-03908	2011	Darss-Zingst Bodden Chain	Seawater	E-BS-sw	LAGuS
VN-03911	2011	Darss-Zingst Bodden Chain	Seawater	E-BS-sw	LAGuS
VN-03916	2011	Darss-Zingst Bodden Chain	Sediment	E-BS-sd	LAGuS
VN-03918	2011	Darss-Zingst	Seawater/sediment	E-BS-sw/sd	LAGuS
VN-03939	2011	Darss-Zingst Bodden Chain	Sediment	E-BS-sd	LAGuS
VN-03942	2011	Darss-Zingst	Seawater/sediment	E-BS-sw/sd	LAGuS
VN-03944	2011	Darss-Zingst	Seawater/sediment	E-BS-sw/sd	LAGuS
VN-03949	2011	Darss-Zingst	Seawater/sediment	E-BS-sw/sd	LAGuS
VN-03951	2011	Darss-Zingst	Seawater/sediment	E-BS-sw/sd	LAGuS
VN-03954	2011	Darss-Zingst	Seawater/sediment	E-BS-sw/sd	LAGuS
VN-03955	2011	Darss-Zingst Bodden Chain	Sediment	E-BS-sd	LAGuS
VN-03958	2011	Darss-Zingst	Seawater/sediment	E-BS-sw/sd	LAGuS
VN-03963	2010	Binz	Seawater	E-BS-sw	LAGuS
VN-04241	2013	Graal-Müritz	Seawater	E-BS-sw	AWI
VN-04250	2013	Heringsdorf	Seawater	E-BS-sw	AWI
VN-05169	2011	Rügen	Seawater	E-BS-sw	IFH
VN-05172	2011	Rügen	Seawater	E-BS-sw	IFH
VN-05174	2011	Rügen	Seawater	E-BS-sw	IFH
VN-05176	2011	Rügen	Seawater	E-BS-sw	IFH
VN-05177	2011	Rügen	Seawater	E-BS-sw	IFH
VN-05185	2011	Rügen	Seawater	E-BS-sw	IFH
VN-05301	2012	Salzhaff/Pepelow	Seawater	E-BS-sw	IFH

Table continued					
**Strain**	**Year of isolation**	**Geographical origin**	**Source**	**Source code[2]**	**Institutional origin[4]**
**Environmental - North Sea**					
VN-02808	2011	Büsum	Seawater	E-NS-sw	AWI
VN-02825	2011	Speicherkoog	Seawater	E-NS-sw	AWI
VN-02923	2011	Schleswig-Holstein	Seawater	E-NS-sw	AWI
VN-03012	2009-2011	n.s.	Seawater	E-NS-sw	AWI
VN-03213	2014	n.s.	Seawater	E-NS-sw	AWI
VN-03301	2010	Dyksterhusen	Seawater/sediment	E-NS-sw/sd	KLIWAS
VN-03361	2010	n.s.	Seawater/sediment	E-NS-sw/sd	KLIWAS
VN-03377	2010	Neßmersiel	Seawater/sediment	E-NS-sw/sd	KLIWAS
VN-03405	2010	Dyksterhusen	Seawater/sediment	E-NS-sw/sd	KLIWAS
VN-03407	2010	Dyksterhusen	Seawater/sediment	E-NS-sw/sd	KLIWAS
VN-03428	2010	Norddeich	Seawater/sediment	E-NS-sw/sd	KLIWAS
VN-03460	2010	Dedesdorf	Seawater/sediment	E-NS-sw/sd	KLIWAS
VN-03469	2010	Dorum	Seawater/sediment	E-NS-sw/sd	KLIWAS
VN-03470	2010	Dorum	Seawater/sediment	E-NS-sw/sd	KLIWAS
VN-03471	2010	Dorum	Seawater/sediment	E-NS-sw/sd	KLIWAS
VN-03472	2010	Dorum	Seawater/sediment	E-NS-sw/sd	KLIWAS
VN-03475	2010	Wremen	Seawater/sediment	E-NS-sw/sd	KLIWAS
VN-03492	2011	n.s.	Seawater/sediment	E-NS-sw/sd	KLIWAS
VN-03503	2009	n.s.	Seawater/sediment	E-NS-sw/sd	KLIWAS
VN-04219	2013	Emden	Seawater	E-NS-sw	AWI
VN-04223	2013	Wangerland	Seawater	E-NS-sw	AWI
VN-04226	2013	Wilhelmshaven	Seawater	E-NS-sw	AWI
VN-04231	2013	Emden	Seawater	E-NS-sw	AWI
VN-04233	2013	Cuxhaven	Seawater	E-NS-sw	AWI
VN-10012	2011	Lower Saxony Wadden Sea	Oyster	E-NS-bm	IFF
VN-10013	2011	Lower Saxony Wadden Sea	Oyster	E-NS-bm	IFF
VN-10127	2012	Lower Saxony Wadden Sea	Oyster[1]	E-NS-bm	IFF
VN-10130	2012	n.s.	Oyster[1]	E-NS-bm	IFF
VN-10131	2012	n.s.	Oyster[1]	E-NS-bm	IFF
VN-10133	2012	n.s.	Oyster[1]	E-NS-bm	IFF
VN-10137	2012	n.s.	Blue mussel	E-NS-bm	IFF
VN-10143	2012	Lower Saxony Wadden Sea	Blue mussel[1]	E-NS-bm	IFF
VN-10144	2012	Lower Saxony Wadden Sea	Blue mussel[1]	E-NS-bm	IFF
VN-10145	2012	Lower Saxony Wadden Sea	Blue mussel[1]	E-NS-bm	IFF
VN-10146	2012	Lower Saxony Wadden Sea	Blue mussel[1]	E-NS-bm	IFF
VN-10150	2012	Lower Saxony Wadden Sea	Blue mussel[1]	E-NS-bm	IFF
VN-10156	2012	Lower Saxony Wadden Sea	Blue mussel	E-NS-bm	IFF
VN-10159	2012	Lower Saxony Wadden Sea	Blue mussel	E-NS-bm	IFF
VN-10162	2012	Lower Saxony Wadden Sea	Blue mussel	E-NS-bm	IFF
VN-10191	2013	Lower Saxony Wadden Sea	Blue mussel[1]	E-NS-bm	IFF
VN-10192	2013	Lower Saxony Wadden Sea	Blue mussel[1]	E-NS-bm	IFF
VN-10196	2013	Lower Saxony Wadden Sea	Blue mussel[1]	E-NS-bm	IFF
VN-10197	2013	Lower Saxony Wadden Sea	Blue mussel[1]	E-NS-bm	IFF
VN-10198	2013	Lower Saxony Wadden Sea	Blue mussel[1]	E-NS-bm	IFF
VN-10204	2013	Lower Saxony Wadden Sea	Oyster[1]	E-NS-bm	IFF
VN-10205	2013	Lower Saxony Wadden Sea	Oyster[1]	E-NS-bm	IFF
VN-10206	2013	Lower Saxony Wadden Sea	Oyster[1]	E-NS-bm	IFF
VN-10207	2013	Lower Saxony Wadden Sea	Oyster[1]	E-NS-bm	IFF
VN-10208	2013	Lower Saxony Wadden Sea	Oyster[1]	E-NS-bm	IFF
VN-10320	2014	Lower Saxony Wadden Sea	Oyster[1]	E-NS-bm	IFF

Table continued					
**Strain**	**Year of isolation**	**Geographical origin**	**Type of infection**	**Source code[2]**	**Institutional origin**
**Clinical - Germany**					
VN-00168	2010	Baltic Sea	Wound infection[3]	C-G-ext	LAGuS
VN-00169	2010	Baltic Sea	Wound infection[3]	C-G-ext	LAGuS
VN-00297	1995	Baltic Sea/Szczecin Lagoon	Otitis	C-G-ext	BfR
VN-00298	1995	Baltic Sea	Otitis	C-G-ext	BfR
VN-00300	1999	n.s.	Diarrhea, peritonitis	C-G-int	BfR
VN-00302	2012	North Sea/Bremerhaven	Diarrhea	C-G-int	RKI
VN-00305	2012	n.s.	Otitis	C-G-ext	RKI
VN-00307	2012	n.s.	Otitis	C-G-ext	RKI
VN-00533	2016	Baltic Sea/Ueckermünde Lagoon	Wound infection	C-G-ext	BfR
VN-00534	2017	Baltic Sea/Greifswald	Otitis	C-G-ext	BfR

AWI: Alfred Wegener Institute, Heligoland, Germany
BfR: Federal Institute for Risk Assessment, Berlin, Germany
IFF: Institute for Fish and Fishery Products, Cuxhaven, Germany (LAVES: Lower Saxony State Office for Consumer Protection and Food Safety)
IFH: Institute of Food Safety and Food Hygiene, Department of Veterinary Medicine, Free University of Berlin, Germany
KLIWAS: Research program "Impacts of climate change on waterways and navigation"
LAGuS: State Office for Health and Social Affairs, Rostock, Germany
RKI: Robert Koch Institute, Berlin, Germany

n.s., not specified.

[1] Organism from cultivation of bivalve mollusks (primary production).
[2] The source code is explained in Table 1.
[3] Isolates from the same patient.
[4] Environmental strains were isolated within the German research programs VibrioNet (AWI, IFF, IFH, LAGuS) and KLIWAS.
