# Supplementary material for: Environmental and Clinical Strains of Vibrio cholerae Non-O1, Non-O139 From Germany Possess Similar Virulence Gene Profiles
Source: Front Microbiol. 2019 Apr 12;10:733. doi: 10.3389/fmicb.2019.00733 (PMC6474259; doi:10.3389/fmicb.2019.00733)
Supplement: Supplementary file 2 [file Table_2.pdf]

Table S2. Primers used for species confirmation, multilocus sequence typing (MLST), and PCR typing of virulence genes.

Gene/Target	Primer	Sequence (5‘ to 3‘)	Amplicon size (bp)	T_a (°C)	References
**Species confirmation, characterization and subtyping**					
toxR	UtoxF	GASTTTGTTTGGCGYGARCAAGGTT	640	59	Bauer et al. (2007)
	VctoxR	GGTTAGCAACGATGCGTAAG			
ctxA	ctxA1	CTCAGACGGGATTTGTTAGGCACG	301		Shirai et al. (1991);
	ctxA2	TCTATCTCTGTAGCCCCTATTACG			Chatterjee et al. (2009)
rfb O1 cluster	O1F	GTTTCACTGAACAGATGGG	192		Hoshino et al. (1998);
	O1R	GGTCATCTGTAAGTACAAC			Mantri et al. (2006);
rfb O139 cluster	O139F	AGCCTCTTTATTACGGGTGG	449		Shuan et al. (2009)
	O139R	GTCAAACCCGATCGTAAAGG			
**Multilocus Sequence Typing (MLST)**					
adk	V-chol-adk F	CATCATTCTTCTCGGTGCTC	592	59	Octavia et al. (2013)
	V-chol-adk R	AGTGCCGTCAAACTTCAGGTA			
gyrB	V-chol-gyrB F	GTACGTTTCTGGCCTAGTGC	749	58	Octavia et al. (2013)
	V-chol-gyrB R	GGGTCTTTTTCCTGACAATC			
mdh	V-chol-mdh F	ATGAAAGTCGCTGTTATTGG			Octavia et al. (2013)
	V-chol-mdh R1[a]	GCCGCTTGGCCCATAGAAAG	692	62	
	V-chol-mdh R2	TAGCTTGATAGGTTGGG	810	51	
metE	V-chol-metE F	CGGGTGACTTTGCTTGGT	827	56	Octavia et al. (2013)
	V-chol-metE R	CAGATCGACTGGGCTGTG			
pntA	V-chol-pntA F1[a]	CTTTGATGGAAAAACTCTCA	740	52	Octavia et al. (2013)
	V-chol-pntA F2	GGCCAGCCCAAAATCCT	758	52	
	V-chol-pntA R	GATATTGCCGTCTTTTTCTT			
purM	V-chol-purM F	GGTGTCGATATTGATGCAGG	734	58	Octavia et al. (2013)
	V-chol-purM R	GGAATGTTTTCCCAGAAGCC			
pyrC	V-chol-pyrC F	ATCATGCCTAACACGGTTCC	726	57	Octavia et al. (2013)
	V-chol-pyrC R	TTCAAACACTTCGGCATA			
**PCR typing of virulence genes**					
chxA	VC-chxA-F	TGTGTGATGATGCTTCTGG	2000	52	Awasthi et al. (2013)
	VC-chxA-R	TTATTTCAGTTCATCTTTTCGC			
	VC-Cholix-fo	GCAACAACACTGAAAACGAG	397	56	This study
	VC-Cholix-re	TCCTCATCAATCGCCAATTC			
$hlyA^{CL/ET}$	hlyA-489F	GGCAAACAGCGAAACAAATACC	727/738	60	Rivera et al. (2001);
$hlyA^{ET}$	hlyA-744F	GAGCCGGCATTCATCTGAAT	481		Singh et al. (2001)
$hlyA^{CL/ET}$	hlyA-1184R	CTCAGCGGGCTAATACGGTTTA			
mshA	VC0409-F	ATTCTCGGTATCTTGGCCGTC	459	62	Rahman et al. (2008)
	VC0409-R	ACAAGCAGTTCCAGCAACCC			

Table continued					
**Gene/Target**	**Primer**	**Sequence (5‘ to 3‘)**	**Amplicon size (bp)**	**T_a (°C)**	**References**
**PCR typing of virulence genes**					
ompU	ompU-F	ACGCTGACGGAATCAACCAAAG	869	60	Rivera et al. (2001);
	ompU-R	GCGGAAGTTTGGCTTGAAGTAG			Singh et al. (2001)
rstR^{Calc}	VC-rstR-calc-F	CCAGCATTTCTGTTTCTTTG	104	56	Rahman et al. (2008)
	VC-rstR-calc-R	GGCAACAAAGCACATTAAAG			
rstR^{CL}	VC-rstR-class-F	CTCATCAGCAAAGCCTCCATC	241	62	Rahman et al. (2008)
	VC-rstR-class-R	TAGCAAATGGTATCGGCGTTG			
rstR^{ET}	VC1455-F	AGCCAACCAAAGAAAGGCAAT	186	62	Rahman et al. (2008)
	VC1455-R	TCATCTGTGGCCCATCTTCC			
rtxA[b]	VC1451-F	GATTCTTCCGTTCAAGCTCCG	2571	63	Schirmeister et al. (2014)
	VC1451-R	TGGTTCAGGCTGTTGCACAC			
rtxC	VC1450-F	TGCAAATCTCACATTAGCGCA	430	63	Schirmeister et al. (2014)
	VC1450-R	CCACTGCACCTTTCGGATACA			
tcpA$^{CL/ET}$	tcpA-F_Class-ET	CACGATAAGAAAACCGGTCAAGAG		60	Mantri et al. (2006)
tcpA^{CL}	tcpA-R_class	TTACCAAATGCAACGCCGAATG	620		
tcpA^{ET}	tcpA-R_ET	AATCATGAGTTCAGCTTCCCGC	823		
TTSS *vcsC2*	TTSS_vcsC2-A	CGTTACCGATGCTATGGGT	535	60	Chatterjee et al. (2009)
	TTSS_vcsC2-B	AGAAGTCGGTTGTTTCGGTAA			
TTSS *vcsN2*	TTSS_vcsN2-A	CAGTTGAGCCAATTCCATT	484	55	Chatterjee et al. (2009)
	TTSS_vcsN2-B	GACCAAACGAGATAATG			
TTSS *vcsV2*	TTSS_vcsV2-A	TTTGGCTCACTTGATGGG	742	55	Chatterjee et al. (2009);
	TTSS_vcsV2-B	GCCACATCATTGCTTGCT			Dziejman et al. (2005)
TTSS *vspD*	TTSS_vspD-A	AACTCGAAGAGCAGAAAAAAGC	422	55	Chatterjee et al. (2009);
	TTSS_vspD-B	CTTCCCGCTTTTGATGAAATG			Dziejman et al. (2005)
VSP-1	Vch-VspI-fo	GCTCTCGCCAGCAAGGAGCTG	1700	60	Rahman et al. (2008)
	Vch-VspI-re	CCGTCGAAGTGAACGGCGAAC			
VSP-2	Vch-VspII-fo	TGCCCATTCCGCTAAGTGTTC	800	60	Rahman et al. (2008)
	Vch-VspII-re	GCAAAAGCACTGCGTAAACTG			

Calc, Calcutta; CL, Classical; ET, El Tor; T_a, annealing temperature.

[a] alternative primer

[b] VC1451 of *Vibrio cholerae* O1 biovar El Tor str. N16961.

References

Awasthi, S. P., Asakura, M., Chowdhury, N., Neogi, S. B., Hinenoya, A., Golbar, H. M., et al. (2013). Novel cholix toxin variants, ADP-ribosylating toxins in *Vibrio cholerae* non-O1/non-O139 strains, and their pathogenicity. *Infect. Immun.* 81, 531-541. doi: 10.1128/IAI.00982-12

Bauer, A. and Rørvik, L. M. (2007). A novel multiplex PCR for the identification of *Vibrio parahaemolyticus*, *Vibrio cholerae* and *Vibrio vulnificus*. *Lett. Appl. Microbiol.* 45, 371-375. doi: 10.1111/j.1472-765X.2007.02195.x

Chatterjee, S., Ghosh, K., Raychoudhuri, A., Chowdhury, G., Bhattacharya, M. K., Mukhopadhyay, A. K., et al. (2009). Incidence, virulence factors, and clonality among clinical strains of non-O1, non-O139 *Vibrio cholerae* isolates from hospitalized diarrheal patients in Kolkata, India. *J. Clin. Microbiol.* 47, 1087-1095. doi: 10.1128/JCM.02026-08

Dziejman, M., Serruto, D., Tam, V. C., Sturtevant, D., Diraphat, P., Faruque, S. M., et al. (2005). Genomic characterization of non-O1, non-O139 *Vibrio cholerae* reveals genes for a type III secretion system. *Proc. Natl. Acad. Sci. U.S.A.* 102, 3465-3470. doi: 10.1073/pnas.0409918102

Hoshino, K., Yamasaki, S., Mukhopadhyay, A. K., Chakraborty, S., Basu, A., Bhattacharya, S. K., et al. (1998). Development and evaluation of a multiplex PCR assay for rapid detection of toxigenic *Vibrio cholerae* O1 and O139. *FEMS Immunol. Med. Microbiol.* 20, 201-207. doi: 10.1111/j.1574-695X.1998.tb01128.x

Mantri, C. K., Mohapatra, S. S., Ramamurthy, T., Ghosh, R., Colwell, R. R., and Singh, D. V. (2006). Septaplex PCR assay for rapid identification of *Vibrio cholerae* including detection of virulence and *int* SXT genes. *FEMS Microbiol. Lett.* 265, 208-214. doi: 10.1111/j.1574-6968.2006.00491.x

Octavia, S., Salim, A., Kurniawan, J., Lam, C., Leung, Q., Ahsan, S., et al. (2013). Population structure and evolution of non-O1/non-O139 *Vibrio cholerae* by multilocus sequence typing. *PLoS ONE* 8:e65342. doi: 10.1371/journal.pone.0065342

Rahman, M. H., Biswas, K., Hossain, M. A., Sack, R. B., Mekalanos, J. J., and Faruque, S. M. (2008). Distribution of genes for virulence and ecological fitness among diverse *Vibrio cholerae* population in a cholera endemic area: tracking the evolution of pathogenic strains. *DNA Cell Biol.* 27, 347-355. doi: 10.1089/dna.2008.0737

Rivera, I. N. G., Chun, J., Huq, A., Sack, R. B., and Colwell, R. R. (2001). Genotypes associated with virulence in environmental isolates of *Vibrio cholerae*. *Appl. Environ. Microbiol.* 67, 2421-2429. doi: 10.1128/AEM.67.6.2421–2429.2001

Schirmeister, F., Dieckmann, R., Bechlars, S., Bier, N., Faruque, S. M., and Strauch, E. (2014). Genetic and phenotypic analysis of *Vibrio cholerae* non-O1, non-O139 isolated from German and Austrian patients. *Eur. J. Clin. Microbiol. Infect. Dis.* 33, 767-778. doi: 10.1007/s10096-013-2011-9

Shirai, H., Nishibuchi, M., Ramamurthy, T., Bhattacharya, S. K., Pal, S. C., and Takeda, Y. (1991). Polymerase chain reaction for detection of the cholera enterotoxin operon of *Vibrio cholerae*. *J. Clin. Microbiol.* 29, 2517-2521.

Shuan Ju Teh, C., Lin Thong, K., Tein Ngoi, S., Ahmad, N., Balakrish Nair, G., and Ramamurthy, T. (2009). Molecular characterization of serogrouping and virulence genes of Malaysian *Vibrio cholerae* isolated from different sources. *J. Gen. Appl. Microbiol.* 55, 419-425. doi: 10.2323/jgam.55.419

Singh, D. V., Matte, M. H., Matte, G. R., Jiang, S., Sabeena, F., Shukla, B. N., et al. (2001). Molecular analysis of *Vibrio cholerae* O1, O139, non-O1, and non-O139 strains: clonal relationships between clinical and environmental isolates. *Appl. Environ. Microbiol.* 67, 910-921. doi: 10.1128/AEM.67.2.910–921.2001
