# Supplementary material for: Environmental and Clinical Strains of Vibrio cholerae Non-O1, Non-O139 From Germany Possess Similar Virulence Gene Profiles
Source: Front Microbiol. 2019 Apr 12;10:733. doi: 10.3389/fmicb.2019.00733 (PMC6474259; doi:10.3389/fmicb.2019.00733)
Supplement: Supplementary file 3 [file Table_3.pdf]

Table S3. Criteria for the interpretation of hemolytic activity data of *Vibrio cholerae* non-O1, non-O139 isolates from German coastal waters analyzed in this study.

Mean diameter of the hemolytic halo (mm)	Category[a]
No hemolysis	-
Hemolysis only under the macrocolony or ≤ 1	+
> 1 and < 4	++
≥ 4	+++

[a] (-), non-hemolytic; (+), weakly hemolytic; (++), intermediately hemolytic; (+++), strongly hemolytic.
