# Supplementary material for: Environmental and Clinical Strains of Vibrio cholerae Non-O1, Non-O139 From Germany Possess Similar Virulence Gene Profiles
Source: Front Microbiol. 2019 Apr 12;10:733. doi: 10.3389/fmicb.2019.00733 (PMC6474259; doi:10.3389/fmicb.2019.00733)
Supplement: Supplementary file 4 [file Table_4.pdf]

Table S4. Clinical *Vibrio cholerae* strains used as amplification controls in PCR typing of virulence genes.

Strain	Species	Year	Geographical origin	Type of infection	Reference	Genes/Features[1]
VN-00211	*Vibrio cholerae* non-O1, non-O139	2011	Italy	Diarrhea	RKI	*rtxA*[2]
VN-00300	*Vibrio cholerae* non-O1, non-O139	1999	Germany	Diarrhea, peritonitis	BfR	*chxA*
VN-00303	*Vibrio cholerae* non-O1, non-O139	2012	Hungary	Otitis	RKI	negative for *hlyA*^{CL}, *hlyA*^{ET}
VN-00308	*Vibrio cholerae* non-O1, non-O139	2012	Romania	Diarrhea	RKI	TTSS *vcsC2*/*vcsN2*/*vspD*/*vcsV2*; *rtxC*
VN-00315	*Vibrio cholerae* non-O1, non-O139	2011	Cuba	Diarrhea	AGES	VSP-2
1576	*Vibrio cholerae* O1	1959	Thailand	Diarrhea	BfR	classical biotype; *rstR*^{CL}
1360	*Vibrio cholerae* O1	1959	Thailand	Diarrhea	BfR	classical biotype; *toxR*; *ctxA*; O1 *rfb*; *hlyA*^{CL}; *ompU*; *tcpA*^{CL}, *tcpA*^{ET}; negative for VSP-1, VSP-2
MO45	*Vibrio cholerae* O139	1992	India	Diarrhea	BfR	El Tor biotype; *toxR*; *ctxA*; O139 *rfb*; *hlyA*^{ET}; *mshA*; *tcpA*^{ET}; VSP-1; *rstR*^{ET}

AGES: Austrian Agency for Health and Food Safety, Vienna, Austria
BfR: Federal Institute for Risk Assessment, Berlin, Germany
RKI: Robert Koch Institute, Berlin, Germany

CL, Classical; ET, El Tor.

[1] Only genes/features relevant to PCR typing of virulence genes are given.
[2] VC1451 of *Vibrio cholerae* O1 biovar El Tor str. N16961.
