# Supplementary material for: Environmental and Clinical Strains of Vibrio cholerae Non-O1, Non-O139 From Germany Possess Similar Virulence Gene Profiles
Source: Front Microbiol. 2019 Apr 12;10:733. doi: 10.3389/fmicb.2019.00733 (PMC6474259; doi:10.3389/fmicb.2019.00733)
Supplement: Supplementary file 5 [file Table_5.pdf]

Table S5. MLST allelic profiles and resulting sequence types of *Vibrio cholerae* non-O1, non-O139 isolates from German coastal waters and from German clinical samples analyzed in this study.[a]

Strain	Source code[b]	Allelic profile							Sequence type
		adk	*gyrB*	*mdh*	*metE*	*pntA*	*purM*	*pyrC*	
**Environmental - Baltic Sea**									
VN-00278	E-BS-sw	**68**	**73**	14	**131**	**84**	**62**	**94**	**488**
VN-00455	E-BS-sw	**60**	**75**	**69**	**120**	**87**	1	n.d.[c]	n.d.
VN-00456	E-BS-sw	**72**	38	14	**126**	**79**	**60**	**92**	**497**
VN-00457	E-BS-sw	48	**84**	46	**127**	**88**	1	**105**	**467**
VN-00458	E-BS-sw	14	30	**66**	91	66	41	73	**454**
VN-00459	E-BS-sw	18	61	**67**	**156**	71	1	35	330
VN-00460	E-BS-sw	3	48	29	36	25	1	12[d]	**445**
VN-00461	E-BS-sw	**72**	**65**	11	**113**	**91**	**60**	**92**	**498**
VN-00462	E-BS-sw	**70**	36	14	**117**	**85**	**60**	**102**	**492**
VN-00463	E-BS-sw	**60**	**70**	**69**	**122**	**87**	1	**113**	**475**
VN-00464	E-BS-sw	**61**	55	14	**146**	**75**	1	**94**	**479**
VN-00465	E-BS-sw	14	49	15	47	**77**	1	**120**	**455**
VN-00466	E-BS-sw	2	23	57	9	59	1	35	**441**
VN-00468	E-BS-sw	**58**	48	**73**	**139**	47	**63**	**90**	**472**
VN-00469	E-BS-sw	**78**	**86**	14	**138**	1	**52**	**115**	**504**
VN-00470	E-BS-sw	**72**	**65**	45	**113**	**91**	**60**	**92**	**499**
VN-00471	E-BS-sw	**70**	36	14	**117**	**85**	**60**	**102**	**492**
VN-00472	E-BS-sw	**70**	36	14	**117**	**85**	**60**	**102**	**492**
VN-00473	E-BS-sw	**70**	**65**	14	**129**	**92**	**60**	**102**	**493**
VN-00474	E-BS-sw	**66**	**86**	14	**143**	47	1	**92**	**485**
VN-00475	E-BS-sw	49	1	**74**	**147**	**93**	**56**	**119**	**468**
VN-00476	E-BS-sw	14	5	14	**123**	**84**	**60**	**91**	**456**
VN-00477	E-BS-sw	**60**	**75**	**69**	**120**	**87**	1	n.d.[c]	n.d.
VN-02995	E-BS-sw	**68**	**73**	14	**131**	**84**	**62**	**94**	**488**
VN-03901	E-BS-sw/sd	18	5	14	43	71	**58**	35	**462**
VN-03902	E-BS-sw/sd	38	5	**75**	**152**	62	1	**121**	**465**
VN-03903	E-BS-sw	48	**84**	46	**127**	**88**	1	**105**	**467**
VN-03907	E-BS-sw/sd	3	48	29	36	25	3	12[d]	**446**
VN-03908	E-BS-sw	2	5	14	**122**	**91**	1	**103**[d]	**442**
VN-03911	E-BS-sw	**63**	5	14	**129**	6	1	67	**481**
VN-03916	E-BS-sd	1	38	14	**122**	**91**	**60**	**103**	**433**
VN-03918	E-BS-sw/sd	53	36	7	9	**90**	1	65	**469**
VN-03939	E-BS-sd	**63**	5	14	**129**	6	1	67	**481**
VN-03942	E-BS-sw/sd	**66**	5	8	36	6	1	**92**	**486**
VN-03944	E-BS-sw/sd	14	5	**74**	**137**	5	**64**	**110**	**458**
VN-03949	E-BS-sw/sd	18	5	15	48	17	1	45	**463**
VN-03951	E-BS-sw/sd	**73**	**85**	14	**149**	19	1	**96**	303
VN-03954	E-BS-sw/sd	1	5	14	**116**	6	1	**92**	**434**
VN-03955	E-BS-sd	1	5	14	**142**	6	1	**92**	**435**
VN-03958	E-BS-sw/sd	13	38	11	**148**	64	**59**	**118**	**451**
VN-03963	E-BS-sw	**70**	**65**	**73**	**116**	13	**60**	**117**	**494**
VN-04241	E-BS-sw	18	**81**	14	**130**	**86**	41	43	**464**
VN-04250	E-BS-sw	14	38	14	**117**	47	**60**	**116**	**459**
VN-05169	E-BS-sw	**65**	5	2	**131**	**80**	**60**	**103**	**484**
VN-05172	E-BS-sw	**74**	5	**62**	**129**	6	1	67	**500**
VN-05174	E-BS-sw	53	5	**62**	**129**	6	1	67	**470**
VN-05176	E-BS-sw	**70**	36	14	**117**	**85**	**60**	**102**	**492**
VN-05177	E-BS-sw	13	**77**	14	**151**	66	**61**	**122**	**452**
VN-05185	E-BS-sw	1	**70**	**74**	**157**	38	1	**111**	**436**
VN-05301	E-BS-sw	14	5	14	192	18	**60**	**96**	**460**

Table continued									
**Strain**	**Source code**[b]	**Allelic profile**							**Sequence type**
		adk	***gyrB***	***mdh***	***metE***	***pntA***	***purM***	***pyrC***	
**Environmental - North Sea**									
VN-02808	E-NS-sw	57	76	14	115	18	1	101	336
VN-02825	E-NS-sw	57	76	14	115	18	1	101	336
VN-02923	E-NS-sw	57	76	14	115	18	1	101	336
VN-03012	E-NS-sw	1	5	**74**	**119**	**80**	**53**	**106**	**432**
VN-03213	E-NS-sw	**65**	1	14	**140**	**93**	**62**	**90**	**483**
VN-03301	E-NS-sw/sd	14	**68**	**75**	19	9	**56**	**114**	**457**
VN-03361	E-NS-sw/sd	**76**	**82**	**76**	**150**	**83**	**65**	67	**502**
VN-03377	E-NS-sw/sd	13	5	15	59	**73**	1	67	**449**
VN-03405	E-NS-sw/sd	57	38	**70**	**125**	18	**54**	**95**	**471**
VN-03407	E-NS-sw/sd	57	38	**70**	**125**	18	**54**	**95**	**471**
VN-03428	E-NS-sw/sd	**79**	5	**68**	**159**	**81**	**57**	**109**	**505**
VN-03460	E-NS-sw/sd	1	5	**74**	**119**	**80**	**53**	**106**	**432**
VN-03469	E-NS-sw/sd	1	5	**74**	**119**	**80**	**53**	**106**	**432**
VN-03470	E-NS-sw/sd	13	1	**64**	**144**	5	1	**108**	**450**
VN-03471	E-NS-sw/sd	1	5	**74**	**119**	**80**	**53**	**106**	**432**
VN-03472	E-NS-sw/sd	1	5	**74**	**119**	**80**	**53**	**106**	**432**
VN-03475	E-NS-sw/sd	1	5	**74**	**119**	**80**	**53**	**106**	**432**
VN-03492	E-NS-sw/sd	1	5	**74**	**119**	**80**	**53**	**106**	**432**
VN-03503	E-NS-sw/sd	**77**	38	**65**	**154**	**91**	1	**93**	**503**
VN-04219	E-NS-sw	**58**	**78**	**63**	**128**	61	8	**100**	**473**
VN-04223	E-NS-sw	**69**	41	15	47	**74**	1	83	**489**
VN-04226	E-NS-sw	3	**79**	14	**153**	66	41	**98**	**447**
VN-04231	E-NS-sw	**58**	**78**	**63**	**128**	61	8	**100**	**473**
VN-04233	E-NS-sw	2	38	14	**123**	13	**60**	**103**	**443**
VN-10012	E-NS-bm	**71**	**74**	**76**	**133**	3	9	39	**495**
VN-10013	E-NS-bm	**71**	**74**	**76**	**133**	3	9	39	**495**
VN-10127	E-NS-bm	**66**	5	8	36	6	1	**92**	**486**
VN-10130	E-NS-bm	**60**	**65**	**71**	**136**	**80**	1	**104**	**476**
VN-10131	E-NS-bm	**60**	**65**	**71**	**136**	**80**	1	**104**	**476**
VN-10133	E-NS-bm	**60**	**83**	14	**126**	**76**	**63**	**91**	**477**
VN-10137	E-NS-bm	**65**	1	14	**140**	**93**	**62**	**90**	**483**
VN-10143	E-NS-bm	13	**71**	**62**	**124**	3	9	39	**453**
VN-10144	E-NS-bm	13	**71**	**62**	**124**	3	9	39	**453**
VN-10145	E-NS-bm	13	**71**	**62**	**124**	3	9	39	**453**
VN-10146	E-NS-bm	13	**71**	**62**	**124**	3	9	39	**453**
VN-10150	E-NS-bm	47	1	14	**160**	18	17	66	**466**
VN-10156	E-NS-bm	**60**	36	61	**134**	**82**	**56**	**89**	**478**
VN-10159	E-NS-bm	**60**	36	61	**134**	**82**	**56**	**89**	**478**
VN-10162	E-NS-bm	**60**	36	61	**134**	**82**	**56**	**89**	**478**
VN-10191	E-NS-bm	13	**71**	**62**	**124**	3	9	39	**453**
VN-10192	E-NS-bm	13	**71**	**62**	**124**	3	9	39	**453**
VN-10196	E-NS-bm	**59**	23	14	**135**	3	1	**97**	**474**
VN-10197	E-NS-bm	14	**64**	15	**121**	3	8	**99**	**461**
VN-10198	E-NS-bm	14	**64**	15	**121**	3	8	**99**	**461**
VN-10204	E-NS-bm	**67**	44	14	**132**	66	**55**	**88**	**487**
VN-10205	E-NS-bm	**67**	44	14	**132**	66	**55**	**88**	**487**
VN-10206	E-NS-bm	**67**	44	14	**132**	66	**55**	**88**	**487**
VN-10207	E-NS-bm	**67**	44	14	**132**	66	**55**	**88**	**487**
VN-10208	E-NS-bm	1	**69**	14	1	64	1	**93**	**437**
VN-10320	E-NS-bm	**59**	23	14	**135**	3	1	**97**	**474**

Table continued									
**Strain**	**Source code**[b]	**Allelic profile**							**Sequence type**
		adk	***gyrB***	***mdh***	***metE***	***pntA***	***purM***	***pyrC***	
**Clinical - Germany**									
VN-00168	C-G-ext	**72**	**65**	45	**114**	**91**	**60**	**92**	**496**
VN-00169	C-G-ext	**72**	**65**	45	**114**	**91**	**60**	**92**	**496**
VN-00297	C-G-ext	**70**	**65**	14	**129**	**92**	1	**107**	**490**
VN-00298	C-G-ext	**70**	38	14	**155**	**85**	**60**	**102**	**491**
VN-00300	C-G-int	13	**74**	**76**	**133**	3	9	39	**448**
VN-00302	C-G-int	2	44	11	64	3	8	43	**438**
VN-00305	C-G-ext	**64**	**65**	11	**141**	2	**60**	**112**	**482**
VN-00307	C-G-ext	**70**	**65**	14	**129**	**92**	1	**107**	**490**
VN-00533[e]	C-G-ext	**70**	**65**	14	**218**	5	**60**	**102**	**589**
VN-00534[e]	C-G-ext	**60**	**83**	14	**143**	**76**	**53**	**91**	**590**

n.d., not determined.

[a] Alleles and sequence types that were new to PubMLST database are in bold print.

[b] The source code is explained in Table 1.

[c] PCR amplification of *pyrC* failed.

[d] Fixed maximum length of *Vibrio cholerae pyrC* allele sequences in PubMLST database (449 bp) required the removal of a 6-nt insertion (GCCACA) between nucleotide 23 and 24. The resulting *pyrC* allele is given.

[e] MLST alleles were determined from *de novo* assemblies of genomes with the web-based tool "MLST 1.8" of the Center for Genomic Epidemiology (Larsen et al., 2012). New allele sequences were verified by PCR amplification and partial gene sequencing and finally submitted to the PubMLST database for assignment of new allele numbers (see Material and Methods).

References

Larsen, M. V., Cosentino, S., Rasmussen, S., Friis, C., Hasman, H., Marvig, R. L., et al. (2012). Multilocus sequence typing of total-genome-sequenced bacteria. *J. Clin. Microbiol.* 50, 1355-1361. doi: 10.1128/JCM.06094-11
