# Supplementary material for: Environmental and Clinical Strains of Vibrio cholerae Non-O1, Non-O139 From Germany Possess Similar Virulence Gene Profiles
Source: Front Microbiol. 2019 Apr 12;10:733. doi: 10.3389/fmicb.2019.00733 (PMC6474259; doi:10.3389/fmicb.2019.00733)
Supplement: Supplementary file 6 [file Table_6.pdf]

Table S6. MLST properties of the *Vibrio cholerae* non-O1, non-O139 populations analyzed in this study.

		Total[a] (n = 108)	Environmental[a] (n = 98)	Baltic Sea[a] (n = 48)	North Sea (n = 50)	Clinical (n = 10)	PubMLST[b]
**Alleles**							
adk	n_t (p_t)	32 (13.0%)	31 (13.1%)	22 (14.4%)	18 (14.0%)	6 (14.3%)	105 (9.9%)
	n_n (p_n)	20 (62.5%)	19 (61.3%)	12 (54.5%)	11 (61.1%)	4 (66.7%)	
gyrB	n_t (p_t)	30 (12.2%)	30 (12.7%)	18 (11.8%)	18 (14.0%)	5 (11.9%)	116 (10.9%)
	n_n (p_n)	17 (56.7%)	17 (56.7%)	8 (44.4%)	10 (55.6%)	3 (60.0%)	
mdh	n_t (p_t)	25 (10.2%)	25 (10.5%)	17 (11.1%)	14 (10.9%)	4 (9.5%)	121 (11.4%)
	n_n (p_n)	14 (56.0%)	14 (56.0%)	7 (41.2%)	10 (71.4%)	1 (25.0%)	
metE	n_t (p_t)	56 (22.8%)	51 (21.5%)	30 (19.6%)	25 (19.4%)	8 (19.0%)	270 (25.5%)
	n_n (p_n)	44 (78.6%)	40 (78.4%)	23 (76.7%)	19 (76.0%)	7 (87.5%)	
pntA	n_t (p_t)	38 (15.4%)	37 (15.6%)	28 (18.3%)	18 (14.0%)	7 (16.7%)	154 (14.5%)
	n_n (p_n)	19 (50.0%)	19 (51.4%)	13 (46.4%)	9 (50.0%)	4 (57.1%)	
purM	n_t (p_t)	20 (8.1%)	20 (8.4%)	12 (7.8%)	14 (10.9%)	5 (11.9%)	96 (9.1%)
	n_n (p_n)	14 (70.0%)	14 (70.0%)	9 (75.0%)	9 (64.3%)	2 (40.0%)	
pyrC	n_t (p_t)	45 (18.3%)	43 (18.1%)	26 (17.0%)	22 (17.1%)	7 (16.7%)	198 (18.7%)
	n_n (p_n)	34 (75.6%)	32 (74.4%)	19 (73.1%)	17 (77.3%)	5 (71.4%)	
**total**	n_t (p_t)	246 (100%)	237 (100%)	153 (100%)	129 (100%)	42 (100%)	1060 (100%)
	n_n (p_n)	162 (65.9%)	155 (65.4%)	91 (59.5%)	85 (65.9%)	26 (61.9%)	
**Sequence types**							
	n_t	74	66	42	25	8	718
	n_n (p_n)	71 (95.9%)	63 (95.5%)	40 (95.2%)	24 (96.0%)	8 (100.0%)	

n, number of isolates; n_t, total number of alleles/sequence types; p_t, proportion of n_t of locus X as a percentage of n_t of all loci of the respective *V. cholerae* population; n_n, number of new alleles/ sequence types; p_n, proportion of n_n as a percentage of n_t.

[a] Two *V. cholerae* strains from the Baltic Sea, VN-00455 and VN-00477, were excluded from the analyses due to failing *pyrC* amplification.

[b] PubMLST database as of November 5, 2018.
