# Supplementary material for: Environmental and Clinical Strains of Vibrio cholerae Non-O1, Non-O139 From Germany Possess Similar Virulence Gene Profiles
Source: Front Microbiol. 2019 Apr 12;10:733. doi: 10.3389/fmicb.2019.00733 (PMC6474259; doi:10.3389/fmicb.2019.00733)
Supplement: Supplementary file 7 [file Table_7.pdf]

Table S7. Presence/absence of virulence-associated genes and gene clusters in *Vibrio cholerae* non-O1, non-O139 isolates from German coastal waters and from German clinical samples based on PCR data.

Strain	Source code[a]	Virulence-associated genes and gene clusters																					
		rfb O1	*rfb* O139	*ctxA*	*toxR*	*tcpA* CL	*tcpA* ET	*rstR* CL	*rstR* ET	*rstR* Calc	VSP-1	VSP-2	*hlyA* CL	*hlyA* ET	*mshA*	*ompU*	*rtxA*[g]	*rtxC*	*chxA*	TTSS *vcsC2*	TTSS *vcsN2*	TTSS *vspD*	TTSS *vcsV2*
**Environmental - Baltic Sea**																							
VN-00278	E-BS-sw	-	-	-	+	-	-	-	-	-	-	-	-	+	-	-	-	+	-	-	-	-	-
VN-00455	E-BS-sw	-	-	-	(+)	-	-	-	-	-	(+)[c]	-	-	+	-	+	-	+	-	-	-	-	-
VN-00456	E-BS-sw	-	-	-	+	-	-	-	-	-	(-)[b]	-	-	+	-	+	-	+	-	-	-	-	-
VN-00457	E-BS-sw	-	-	-	+	-	-	-	-	-	(+)[c]	-	-	+	-	-	+	+	-	-	-	-	-
VN-00458	E-BS-sw	-	-	-	+	-	-	-	-	-	(+)[c]	-	-	+	-	+	-	+	+	-	-	-	-
VN-00459	E-BS-sw	-	-	-	+	-	-	-	-	-	-	-	-	(+)[e]	+	+	+	+	-	+	+	+	+
VN-00460	E-BS-sw	-	-	-	+	-	-	-	-	-	-	-	-	+	-	-	-	+	-	-	-	-	-
VN-00461	E-BS-sw	-	-	-	+	-	-	-	-	-	(-)[b]	-	-	+	-	+	-	+	-	-	-	-	-
VN-00462	E-BS-sw	-	-	-	+	-	-	-	-	-	-	-	-	+	-	+	-	+	-	-	-	-	-
VN-00463	E-BS-sw	-	-	-	(+)	-	-	-	-	-	(-)[b]	-	-	(+)[d]	-	+	(+)	+	-	-	-	-	-
VN-00464	E-BS-sw	-	-	-	+	-	-	-	-	-	-	-	-	+	-	+	-	+	-	-	-	-	-
VN-00465	E-BS-sw	-	-	-	+	-	-	-	-	-	(-)[b]	-	-	+	-	-	-	+	-	-	-	-	-
VN-00466	E-BS-sw	-	-	-	+	-	-	-	-	-	-	(+)[c]	-	+	-	+	+	+	+	-	-	-	-
VN-00468	E-BS-sw	-	-	-	+	-	-	-	-	-	(-)[b]	-	-	+	-	+	-	+	-	-	-	-	-
VN-00469	E-BS-sw	-	-	-	+	-	-	-	-	-	-	(+)[c]	-	+	-	+	-	+	-	-	-	-	-
VN-00470	E-BS-sw	-	-	-	+	-	-	-	-	-	(+)[c]	(+)[c]	-	+	-	+	-	+	-	-	-	-	-
VN-00471	E-BS-sw	-	-	-	+	-	-	-	-	-	-	-	-	+	-	+	-	+	-	-	-	-	-
VN-00472	E-BS-sw	-	-	-	+	-	-	-	-	-	-	-	-	+	-	+	-	+	-	-	-	-	-
VN-00473	E-BS-sw	-	-	-	+	-	-	-	-	-	-	-	-	+	-	+	-	+	-	-	-	-	-
VN-00474	E-BS-sw	-	-	-	+	-	-	-	-	-	-	-	-	+	-	+	-	+	-	-	-	-	-
VN-00475	E-BS-sw	-	-	-	+	-	-	-	-	-	(-)[b]	-	-	+	-	+	-	+	-	-	-	-	-
VN-00476	E-BS-sw	-	-	-	+	-	-	-	-	-	-	-	-	+	-	+	-	+	-	-	-	-	-
VN-00477	E-BS-sw	-	-	-	+	-	-	-	-	-	(-)[b]	-	-	+	-	+	-	+	-	-	-	-	-
VN-02995	E-BS-sw	-	-	-	+	-	-	-	-	-	-	-	-	+	-	-	-	+	-	-	-	-	-
VN-03901	E-BS-sw/sd	-	-	-	+	-	-	-	-	-	-	-	-	+	+	+	+	+	-	-	-	-	-
VN-03902	E-BS-sw/sd	-	-	-	+	-	-	-	-	-	(+)[c]	-	-	+	-	(+)	+	+	+	-	-	-	-
VN-03903	E-BS-sw	-	-	-	+	-	-	-	-	-	(+)[c]	-	-	+	-	-	(+)	+	-	-	-	-	-
VN-03907	E-BS-sw/sd	-	-	-	+	-	-	-	-	-	-	-	-	+	-	-	-	+	-	-	-	-	-
VN-03908	E-BS-sw	-	-	-	+	-	-	-	-	-	-	-	-	+	-	+	-	+	-	-	-	-	-
VN-03911	E-BS-sw	-	-	-	+	-	-	-	-	-	(+)[c]	-	-	+	-	-	+	+	-	-	-	-	-
VN-03916	E-BS-sd	-	-	-	+	-	-	-	-	-	-	(+)[c]	-	+	-	+	-	+	-	-	-	-	-
VN-03918	E-BS-sw/sd	-	-	-	+	-	-	-	-	-	-	-	-	+	-	+	-	+	+	-	-	-	-
VN-03939	E-BS-sd	-	-	-	+	-	-	-	-	-	(+)[c]	-	-	+	-	-	+	+	-	-	-	-	-
VN-03942	E-BS-sw/sd	-	-	-	+	-	-	-	-	-	(-)[b]	-	-	+	-	+	-	+	-	-	-	-	-
VN-03944	E-BS-sw/sd	-	-	-	+	-	-	-	-	-	(-)[b]	-	-	+	-	+	-	+	-	-	-	-	-
VN-03949	E-BS-sw/sd	-	-	-	+	-	-	-	-	-	-	-	-	+	-	+	+	+	-	+	+	+	+
VN-03951	E-BS-sw/sd	-	-	-	+	-	-	-	-	-	-	-	-	+	-	-	+	+	-	+	+	+	+
VN-03954	E-BS-sw/sd	-	-	-	+	-	-	-	-	-	(-)[b]	-	-	+	-	+	-	+	-	-	-	-	-
VN-03955	E-BS-sd	-	-	-	+	-	-	-	-	-	(-)[b]	-	-	+	-	+	-	+	-	-	-	-	-
VN-03958	E-BS-sw/sd	-	-	-	+	-	-	-	-	-	-	-	-	+	-	+	-	+	+	-	-	-	-

Table continued																							
**Strain**	**Source code[a]**	**Virulence-associated genes and gene clusters**																					
		***rfb* O1**	***rfb* O139**	***ctxA***	***toxR***	***tcpA* CL**	***tcpA* ET**	***rstR* CL**	***rstR* ET**	***rstR* Calc**	**VSP-1**	**VSP-2**	***hlyA* CL**	***hlyA* ET**	***mshA***	***ompU***	***rtxA*[g]**	***rtxC***	***chxA***	**TTSS *vcsC2***	**TTSS *vcsN2***	**TTSS *vspD***	**TTSS *vcsV2***
VN-03963	E-BS-sw	-	-	-	+	-	-	-	-	-	(+)[c]	-	-	+	-	+	-	+	-	-	-	-	-
VN-04241	E-BS-sw	-	-	-	+	-	-	-	-	-	-	-	-	+	-	+	-	+	+	-	-	-	-
VN-04250	E-BS-sw	-	-	-	+	-	-	-	-	-	(-)[b]	(+)[c]	-	+	+	+	-	+	-	-	-	-	-
VN-05169	E-BS-sw	-	-	-	+	-	-	-	-	-	-	-	-	+	-	+	-	+	-	-	-	-	-
VN-05172	E-BS-sw	-	-	-	+	-	-	-	-	-	-	-	-	+	-	+	+	+	-	-	-	-	-
VN-05174	E-BS-sw	-	-	-	+	-	-	-	-	-	-	-	-	+	-	+	-	+	-	-	-	-	-
VN-05176	E-BS-sw	-	-	-	+	-	-	-	-	-	-	-	-	+	-	+	-	+	-	-	-	-	-
VN-05177	E-BS-sw	-	-	-	+	-	-	-	-	-	(+)[c]	(+)[c]	-	+	-	-	+	+	+	-	-	-	-
VN-05185	E-BS-sw	-	-	-	+	-	-	-	-	-	-	-	-	+	-	-	-	+	-	-	-	-	-
VN-05301	E-BS-sw	-	-	-	+	-	-	-	-	-	-	(+)[c]	-	+	-	-	+	+	-	+	+	+	+
**Environmental - North Sea**																							
VN-02808	E-NS-sw	-	-	-	+	-	-	-	-	-	(+)[c]	-	-	-	-	-	-	(+)	-	-	-	-	-
VN-02825	E-NS-sw	-	-	-	+	-	-	-	-	-	(+)[c]	-	-	-	-	-	-	(+)	-	-	-	-	-
VN-02923	E-NS-sw	-	-	-	+	-	-	-	-	-	(+)[c]	-	-	-	-	-	-	(+)	-	-	-	-	-
VN-03012	E-NS-sw	-	-	-	+	-	-	-	-	-	(-)[b]	-	-	+	-	+	-	+	-	-	-	-	-
VN-03213	E-NS-sw	-	-	-	+	-	-	-	-	-	(+)[c]	-	-	+	-	+	-	+	-	-	-	-	-
VN-03301	E-NS-sw/sd	-	-	-	+	-	-	-	-	-	-	-	-	+	-	-	+	+	+	-	-	-	-
VN-03361	E-NS-sw/sd	-	-	-	+	-	-	-	-	-	(+)[c]	-	-	(+)[e]	-	-	+	+	-	+	+	+	+
VN-03377	E-NS-sw/sd	-	-	-	+	-	-	-	-	-	-	(+)[c]	-	+	-	-	+	+	+	-	-	-	-
VN-03405	E-NS-sw/sd	-	-	-	+	-	-	-	-	-	-	(+)[c]	-	+	-	-	-	+	-	-	-	-	-
VN-03407	E-NS-sw/sd	-	-	-	+	-	-	-	-	-	-	(+)[c]	-	+	-	-	-	+	-	-	-	-	-
VN-03428	E-NS-sw/sd	-	-	-	+	-	-	-	-	-	(+)[c]	-	-	(+)[d]	+	-	-	+	-	-	-	-	-
VN-03460	E-NS-sw/sd	-	-	-	+	-	-	-	-	-	(-)[b]	-	-	+	-	+	-	+	-	-	-	-	-
VN-03469	E-NS-sw/sd	-	-	-	+	-	-	-	-	-	(-)[b]	-	-	+	-	+	-	+	-	-	-	-	-
VN-03470	E-NS-sw/sd	-	-	-	+	-	-	-	-	-	(-)[b]	-	-	+	-	-	-	+	+	-	-	-	-
VN-03471	E-NS-sw/sd	-	-	-	+	-	-	-	-	-	(-)[b]	-	-	+	-	+	-	+	-	-	-	-	-
VN-03472	E-NS-sw/sd	-	-	-	+	-	-	-	-	-	(-)[b]	-	-	+	-	+	-	+	-	-	-	-	-
VN-03475	E-NS-sw/sd	-	-	-	+	-	-	-	-	-	(-)[b]	-	-	+	-	+	-	+	-	-	-	-	-
VN-03492	E-NS-sw/sd	-	-	-	+	-	-	-	-	-	(+)[c]	-	-	+	-	+	-	+	-	-	-	-	-
VN-03503	E-NS-sw/sd	-	-	-	+	-	-	-	-	-	-	-	-	+	-	+	-	+	-	-	-	-	-
VN-04219	E-NS-sw	-	-	-	+	-	-	-	-	-	-	-	-	+	-	+	-	+	+	-	-	-	-
VN-04223	E-NS-sw	-	-	-	+	-	-	-	-	-	(-)[b]	-	-	+	-	(+)	+	+	-	-	-	-	-
VN-04226	E-NS-sw	-	-	-	+	-	-	-	-	-	(+)[c]	-	-	+	-	+	+	+	+	-	-	-	-
VN-04231	E-NS-sw	-	-	-	+	-	-	-	-	-	-	-	-	+	-	+	-	+	+	-	-	-	-
VN-04233	E-NS-sw	-	-	-	+	-	-	-	-	-	-	-	-	+	-	+	-	+	-	-	-	-	-
VN-10012	E-NS-bm	-	-	-	+	-	-	-	-	-	(-)[b]	-	-	+	-	-	-	+	+	-	-	-	-
VN-10013	E-NS-bm	-	-	-	+	-	-	-	-	-	(-)[b]	-	-	+	-	-	-	+	+	-	-	-	-
VN-10127	E-NS-bm	-	-	-	+	-	-	-	-	-	(-)[b]	-	-	+	-	+	-	+	-	-	-	-	-
VN-10130	E-NS-bm	-	-	-	+	-	-	-	-	-	(+)[c]	-	-	+	-	+	-	+	-	-	-	-	-
VN-10131	E-NS-bm	-	-	-	+	-	-	-	-	-	(+)[c]	-	-	+	-	+	-	+	-	-	-	-	-
VN-10133	E-NS-bm	-	-	-	+	-	-	-	-	-	(-)[b]	-	-	+	-	+	-	+	-	-	-	-	-
VN-10137	E-NS-bm	-	-	-	+	-	-	-	-	-	(+)[c]	-	-	+	-	+	-	+	-	-	-	-	-
VN-10143	E-NS-bm	-	-	-	+	-	-	-	-	-	-	(+)[c]	-	+	-	+	-	(+)	+	-	-	-	-
VN-10144	E-NS-bm	-	-	-	+	-	-	-	-	-	-	(+)[c]	-	+	-	+	-	(+)	-	-	-	-	-

Table continued																							
**Strain**	**Source code**[a]	**Virulence-associated genes and gene clusters**																					
		***rfb* O1**	***rfb* O139**	***ctxA***	***toxR***	***tcpA* CL**	***tcpA* ET**	***rstR* CL**	***rstR* ET**	***rstR* Calc**	**VSP-1**	**VSP-2**	***hlyA* CL**	***hlyA* ET**	***mshA***	***ompU***	***rtxA*[g]**	***rtxC***	***chxA***	**TTSS *vcsC2***	**TTSS *vcsN2***	**TTSS *vspD***	**TTSS *vcsV2***
VN-10145	E-NS-bm	-	-	-	+	-	-	-	-	-	-	(+)[c]	-	+	-	+	-	(+)	+	-	-	-	-
VN-10146	E-NS-bm	-	-	-	+	-	-	-	-	-	-	(+)[c]	-	+	-	+	-	(+)	+	-	-	-	-
VN-10150	E-NS-bm	-	-	-	+	-	-	-	-	-	-	-	-	+	-	+	+	+	-	-	-	-	-
VN-10156	E-NS-bm	-	-	-	+	-	-	-	-	-	-	-	-	+	-	+	-	+	-	-	-	-	-
VN-10159	E-NS-bm	-	-	-	+	-	-	-	-	-	-	-	-	+	-	+	-	+	-	-	-	-	-
VN-10162	E-NS-bm	-	-	-	+	-	-	-	-	-	-	-	-	+	-	+	-	+	-	-	-	-	-
VN-10191	E-NS-bm	-	-	-	+	-	-	-	-	-	-	(+)[c]	-	+	-	+	-	(+)	+	-	-	-	-
VN-10192	E-NS-bm	-	-	-	+	-	-	-	-	-	-	(+)[c]	-	+	-	+	-	(+)	+	-	-	-	-
VN-10196	E-NS-bm	-	-	-	+	-	-	-	-	-	-	(+)[c]	-	+	+	+	+	+	-	+	+	+	+
VN-10197	E-NS-bm	-	-	-	+	-	-	-	-	-	-	(+)[c]	-	+	-	+	-	+	+	-	-	-	-
VN-10198	E-NS-bm	-	-	-	+	-	-	-	-	-	-	(+)[c]	-	+	-	+	-	+	+	-	-	-	-
VN-10204	E-NS-bm	-	-	-	+	-	-	-	-	-	(-)[b]	-	-	+	-	(+)	-	+	-	-	-	-	-
VN-10205	E-NS-bm	-	-	-	+	-	-	-	-	-	(-)[b]	-	-	+	-	(+)	-	+	-	-	-	-	-
VN-10206	E-NS-bm	-	-	-	+	-	-	-	-	-	(-)[b]	-	-	+	-	(+)	-	+	-	-	-	-	-
VN-10207	E-NS-bm	-	-	-	+	-	-	-	-	-	(-)[b]	-	-	+	-	(+)	-	+	-	-	-	-	-
VN-10208	E-NS-bm	-	-	-	+	-	-	-	-	-	-	-	-	+	-	+	-	+	-	-	-	-	-
VN-10320	E-NS-bm	-	-	-	+	-	-	-	-	-	-	(+)[c]	-	+	+	+	+	+	-	+	+	+	+
**Clinical - Germany**																							
VN-00168[f]	C-G-ext	-	-	-	+	-	-	-	-	-	(-)[b]	-	-	+	-	+	-	+	-	-	-	-	-
VN-00169[f]	C-G-ext	-	-	-	+	-	-	-	-	-	(-)[b]	-	-	+	-	+	-	+	-	-	-	-	-
VN-00297[f]	C-G-ext	-	-	-	+	-	-	-	-	-	-	-	-	+	-	+	-	+	-	-	-	-	-
VN-00298[f]	C-G-ext	-	-	-	+	-	-	-	-	-	-	-	-	+	-	+	-	+	-	-	-	-	-
VN-00300[f]	C-G-int	-	-	-	+	-	-	-	-	-	(-)[b]	-	-	+	-	+	-	+	+	+	+	+	+
VN-00302[f]	C-G-int	-	-	-	+	-	-	-	-	-	-	-	-	+	-	-	-	+	-	+	+	+	+
VN-00305[f]	C-G-ext	-	-	-	+	-	-	-	-	-	-	-	-	+	-	+	-	+	-	-	-	-	-
VN-00307[f]	C-G-ext	-	-	-	+	-	-	-	-	-	-	-	-	+	-	+	-	+	-	-	-	-	-
VN-00533	C-G-ext	-	-	-	+	-	-	-	-	-	-	-	-	+	-	+	-	+	-	(+)	+	+	+
VN-00534	C-G-ext	-	-	-	+	-	-	-	-	-	(-)[b]	-	-	+	-	-	-	+	-	-	-	-	-

Calc, Calcutta; CL, Classical; ET, El Tor.

[a] The source code is explained in Table 1.

The absence of VSP-1 and VSP-2 is demonstrated by the generation of 1.7 kbp and 800 bp PCR products, respectively, as the primers bind to flanking genomic sites of the pandemic islands.

[b] PCR product of ca. 3 kbp and 4 kbp, respectively.

[c] No visible or unspecific PCR products.

The presence of *hlyA*^{ET} gene is demonstrated by the generation of 738 bp and 481 bp PCR products.

[d] 738 bp PCR product only weakly visible.

[e] 481 bp PCR product only weakly visible.

[f] The presence/absence data of virulence-associated genes (except *chxA*) and gene clusters were obtained from Schirmeister et al. (2014).

[g] VC1451 of *Vibrio cholerae* O1 biovar El Tor str. N16961.

References

Schirmeister, F., Dieckmann, R., Bechlars, S., Bier, N., Faruque, S. M., and Strauch, E. (2014). Genetic and phenotypic analysis of *Vibrio cholerae* non-O1, non-O139 isolated from German and Austrian patients. *Eur. J. Clin. Microbiol. Infect. Dis.* 33, 767-778. doi: 10.1007/s10096-013-2011-9
