# Supplementary material for: Environmental and Clinical Strains of Vibrio cholerae Non-O1, Non-O139 From Germany Possess Similar Virulence Gene Profiles
Source: Front Microbiol. 2019 Apr 12;10:733. doi: 10.3389/fmicb.2019.00733 (PMC6474259; doi:10.3389/fmicb.2019.00733)
Supplement: Supplementary file 8 [file Table_8.pdf]

Table S8. Hemolytic activity of *Vibrio cholerae* non-O1, non-O139 isolates from German coastal waters analyzed in this study.

Strain	Source code[a]	Hemolytic activity[b]	
		Sheep erythrocytes	Human erythrocytes
**Environmental - Baltic Sea**			
VN-00278	E-BS-sw	+++	+++
VN-00455	E-BS-sw	++	++
VN-00456	E-BS-sw	+	+
VN-00457	E-BS-sw	++	++
VN-00458	E-BS-sw	++	++
VN-00459	E-BS-sw	++	++
VN-00460	E-BS-sw	++	++
VN-00461	E-BS-sw	++	++
VN-00462	E-BS-sw	++	++
VN-00463	E-BS-sw	++	++
VN-00464	E-BS-sw	-	++
VN-00465	E-BS-sw	++	++
VN-00466	E-BS-sw	++	++
VN-00468	E-BS-sw	+	++
VN-00469	E-BS-sw	++	++
VN-00470	E-BS-sw	++	++
VN-00471	E-BS-sw	+	++
VN-00472	E-BS-sw	++	++
VN-00473	E-BS-sw	++	++
VN-00474	E-BS-sw	+	++
VN-00475	E-BS-sw	++	++
VN-00476	E-BS-sw	+	++
VN-00477	E-BS-sw	++	++
VN-02995	E-BS-sw	++	++
VN-03901	E-BS-sw/sd	+++	+++
VN-03902	E-BS-sw/sd	++	++
VN-03903	E-BS-sw	++	++
VN-03907	E-BS-sw/sd	++	++
VN-03908	E-BS-sw	+++	+++
VN-03911	E-BS-sw	++	++
VN-03916	E-BS-sd	++	+++
VN-03918	E-BS-sw/sd	++	++
VN-03939	E-BS-sd	+++	+++
VN-03942	E-BS-sw/sd	+	++
VN-03944	E-BS-sw/sd	+++	+++
VN-03949	E-BS-sw/sd	++	++
VN-03951	E-BS-sw/sd	++	+++
VN-03954	E-BS-sw/sd	+	++
VN-03955	E-BS-sd	+	++
VN-03958	E-BS-sw/sd	++	++
VN-03963	E-BS-sw	+++	+++
VN-04241	E-BS-sw	+	++
VN-04250	E-BS-sw	++	++
VN-05169	E-BS-sw	+	+
VN-05172	E-BS-sw	++	+
VN-05174	E-BS-sw	+	++
VN-05176	E-BS-sw	+	++
VN-05177	E-BS-sw	+	++
VN-05185	E-BS-sw	++	++
VN-05301	E-BS-sw	++	++

Table continued			
**Strain**	**Source code[a]**	**Hemolytic activity[b]**	
		**Sheep erythrocytes**	**Human erythrocytes**
**Environmental - North Sea**			
VN-02808	E-NS-sw	-	-
VN-02825	E-NS-sw	-	-
VN-02923	E-NS-sw	-	-
VN-03012	E-NS-sw	++	+++
VN-03213	E-NS-sw	++	++
VN-03301	E-NS-sw/sd	++	++
VN-03361	E-NS-sw/sd	++	++
VN-03377	E-NS-sw/sd	++	++
VN-03405	E-NS-sw/sd	+++	+++
VN-03407	E-NS-sw/sd	+++	+++
VN-03428	E-NS-sw/sd	+	++
VN-03460	E-NS-sw/sd	++	+++
VN-03469	E-NS-sw/sd	++	+++
VN-03470	E-NS-sw/sd	++	++
VN-03471	E-NS-sw/sd	++	+++
VN-03472	E-NS-sw/sd	++	+++
VN-03475	E-NS-sw/sd	++	+++
VN-03492	E-NS-sw/sd	++	+++
VN-03503	E-NS-sw/sd	+	+
VN-04219	E-NS-sw	++	++
VN-04223	E-NS-sw	++	++
VN-04226	E-NS-sw	++	++
VN-04231	E-NS-sw	++	++
VN-04233	E-NS-sw	+	++
VN-10012	E-NS-bm	+	++
VN-10013	E-NS-bm	+	++
VN-10127	E-NS-bm	+	++
VN-10130	E-NS-bm	+	++
VN-10131	E-NS-bm	+	++
VN-10133	E-NS-bm	++	++
VN-10137	E-NS-bm	+++	+++
VN-10143	E-NS-bm	++	++
VN-10144	E-NS-bm	++	++
VN-10145	E-NS-bm	++	++
VN-10146	E-NS-bm	++	++
VN-10150	E-NS-bm	++	++
VN-10156	E-NS-bm	++	++
VN-10159	E-NS-bm	+++	+++
VN-10162	E-NS-bm	++	++
VN-10191	E-NS-bm	+	+
VN-10192	E-NS-bm	++	+
VN-10196	E-NS-bm	+	++
VN-10197	E-NS-bm	++	++
VN-10198	E-NS-bm	++	++
VN-10204	E-NS-bm	+	+
VN-10205	E-NS-bm	+	++
VN-10206	E-NS-bm	+	++
VN-10207	E-NS-bm	++	++
VN-10208	E-NS-bm	++	++
VN-10320	E-NS-bm	++	++

[a] The source code is explained in Table 1.
[b] (-), non-hemolytic; (+), weak; (++), intermediate; (+++), strong. The categories of hemolysis are further explained in Table S3.
