# Supplementary material for: Environmental and Clinical Strains of Vibrio cholerae Non-O1, Non-O139 From Germany Possess Similar Virulence Gene Profiles
Source: Front Microbiol. 2019 Apr 12;10:733. doi: 10.3389/fmicb.2019.00733 (PMC6474259; doi:10.3389/fmicb.2019.00733)
Supplement: Supplementary file 11 [file Image_1.pdf]

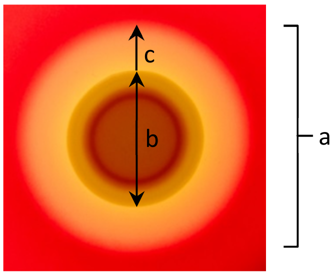

Figure S1. Elicitation of hemolytic activity data for *Vibrio cholerae* non-O1, non-O139 isolates from German coastal waters analyzed in this study (photograph: BfR, Friedmann-Marohn, 2015). Overnight cultures of *V. cholerae* non-O1, non-O139 strains were spotted on Mueller-Hinton agar containing 4% human erythrocytes or 4% sheep erythrocytes. After incubation at 37°C for 22-24 h, the hemolytic activity was characterized based on the diameter of the hemolysis zone around the macrocolony. To minimize measuring inaccuracies sometimes resulting from hemolytic halos without sharply defined edges, the diameter of the hemolysis zone (a) and the diameter of the macrocolony (b) were measured at the bottom of the Petri dish against the light and the mean diameter of the hemolytic halo (c) was mathematically calculated in millimeters [$\frac{(\boldsymbol{a}-\boldsymbol{b})}{\mathbf{2}} = \boldsymbol{c}$]. Hemolysis zones also included parts on the outer edge of the hemolytic halo with incomplete lysis. Further details are given in Material and Methods.
